# Chromosome conformation maps in fission yeast reveal cell cycle dependent sub nuclear structure

**DOI:** 10.1093/nar/gku965

**Published:** 2014-10-23

**Authors:** Ralph S. Grand, Tatyana Pichugina, Lutz R. Gehlen, M. Beatrix Jones, Peter Tsai, Jane R. Allison, Robert Martienssen, Justin M. O'Sullivan

**Affiliations:** 1 Liggins institute, University of Auckland, Grafton Auckland 1032, NZ; 2 Institute of Natural and Mathematical Sciences, Massey University, Albany, Auckland 0745, NZ; 3 School of Biological Sciences, University of Auckland, Auckland 1023, NZ; 4 HHMI-GBMF, Watson School of Biological Sciences, Cold Spring Harbor Laboratory, 1 Bungtown Road, Cold Spring Harbor, New York, NY 11724, USA

## Abstract

Successful progression through the cell cycle requires spatial and temporal regulation of gene transcript levels and the number, positions and condensation levels of chromosomes. Here we present a high resolution survey of genome interactions in *Schizosaccharomyces pombe* using synchronized cells to investigate cell cycle dependent changes in genome organization and transcription. Cell cycle dependent interactions were captured between and within *S. pombe* chromosomes. Known features of genome organization (e.g. the clustering of telomeres and retrotransposon long terminal repeats (LTRs)) were observed throughout the cell cycle. There were clear correlations between transcript levels and chromosomal interactions between genes, consistent with a role for interactions in transcriptional regulation at specific stages of the cell cycle. *In silico* reconstructions of the chromosome organization within the *S. pombe* nuclei were made by polymer modeling. These models suggest that groups of genes with high and low, or differentially regulated transcript levels have preferred positions within the *S. pombe* nucleus. We conclude that the *S. pombe* nucleus is spatially divided into functional sub-nuclear domains that correlate with gene activity. The observation that chromosomal interactions are maintained even when chromosomes are fully condensed in M phase implicates genome organization in epigenetic inheritance and bookmarking.

## INTRODUCTION

The spatial and temporal organization of the genome are increasingly recognized as key contributors to genome maintenance and gene regulation in both prokaryotes and eukaryotes (1–5). High resolution microscopy and proximity based ligation techniques are beginning to reveal how genomes are organized in three-dimensional (3D) space and how this organization relates to genome function (1,6–12). In particular observations that: (i) eukaryotic chromosomes exist in territories (13); (ii) topologically associated domains (TADs) form within chromosomes (12,14,15); (iii) transcription and replication factories form within nuclei (e.g. (16)); and (iv) highly transcribed genes associate in space (8), are thought to be important for the translation of the genotype into the cell's phenotype.

The 3D organization of a genome is the sum of the interplay between the biophysical characteristics of the DNA polymer, DNA packaging and the nuclear processes that are occurring at any specific moment in time. DNA replication and cell growth are key factors that affect the 3D organization of the genome. Cell growth proceeds in an ordered manner through a regulated cycle consisting of the gap 1 (G1), synthesis (S), gap 2 (G2) and mitotic (M) phases. The complexity of cell cycle regulation and large genome sizes make it difficult to interrogate the relationship between genome spatial organization and function through the metazoan cell cycle. Despite this, Naumova *et al.* succeeded in interrogating the intrachromosomal organization, focusing on the mitotic phase structures, of particular chromosomes in human HeLaS3, K562 and primary human foreskin fibroblast cells (12). They observed high levels of correlation between the intrachromosomal organization patterns for early G1, mid G1 and S phase chromosomes (12) and found that mitotic chromosomes maintain few of the structural features that define interphase chromosomes.

The small size of the fission yeast genome and the ability to manipulate the cell cycle makes *Schizosaccharomyces pombe* amenable to studies into the relationship between spatial and functional genome organization through the cell cycle (17–19). In addition, *S. pombe* shares many mammalian features including linear chromosomes, constitutive pericentromeric and telomeric heterochromatin, and cell division by medial fission. As for higher eukaryotes, chromosomal territories and associations among highly transcribed genes have been observed in unsynchronized populations of *S. pombe* cells (8). Moreover, there is increasing evidence that the spatial organization of the *S. pombe* genome is linked with transcriptional activity (1–5). However, it is known that heterochromatin formation (20), and clustering of telomeres, centromeres, mating type loci (21,22) and gene transcript levels fluctuate throughout the *S. pombe* cell cycle (23,24). Therefore, the use of asynchronous cells in studies of *S. pombe* genome organization means that the role cell cycle-specific variations in the 3D arrangement of the genome plays in gene regulation remains unresolved (8).

Here we present the first high resolution analyses of 3D genome organization for populations of fission yeast cells synchronized in the G1, G2 and mitotic anaphase (hereafter M phase), allowing us to infer dynamic connections between and within chromosomes through the cell cycle. Moreover, specific subsets of these interactions are correlated with waves of transcriptional activity between the cell cycle phases. Polymer models of the genome organization in the G1 and G2 cell cycle phases demonstrate that these correlations extend to sub-nuclear localization. Collectively, our results implicate genome organization in epigenetic inheritance and bookmarking.

## MATERIALS AND METHODS

### Strains, growth conditions and synchronization

*Schizosaccharomyces pombe* strains MY291 (h- lue1 cdc10–129), MY284 (h- lue1 cdc25–220) and MY286 (h- lue1 nuc2–663) (Supplementary Table S1) were recovered from −80°C on YES (25) (2% agar) plates (26°C, 4 days). YES medium (12 ml) starter cultures were inoculated and incubated (26°C, 200 rpm) until the OD_595_ measured ∼0.8 (∼24 h). Synchronization cultures (125 ml EMM2 (25), in baffled flasks) were inoculated with starter culture to an OD_595_ = ∼0.05 and incubated (26°C, 120 rpm). Cultures were grown for four generations (OD_595_ ∼0.8) before synchronization was induced by the addition of pre-warmed EMM2 medium (125 ml, 46°C), instantly raising the temperature of the culture to the restrictive temperature (36°C). Cultures were incubated in a hot water bath (36°C, 140 rpm, for 4 h) to complete synchronization. Cells for synchronization efficiency analysis were harvested from cultures before induction and following synchronization (1 ml, 4000 rpm, 2 min), and snap frozen (dry ice/ethanol (100%) bath).

### Synchronization efficiency

Cells collected during synchronization were thawed, washed once with ice-cold 1% phosphate buffered saline (PBS) (500 μl, 4000 rpm, 2 min) and suspended in PBS (100 μl). Cells were stained with calcofluor white (1g/l with 10% Potassium Hydroxide) and 4',6-diamidino-2-phenylindole (DAPI) (25 mg/ml) and photographs were taken of each sample before and following synchronization using a fluorescence microscope (ZEISS, HBO 100 Axiostart plus). The level of cell cycle phase synchronization was calculated for the G1 and G2 phases by comparing the proportion of cells that had a septum, in >200 cells, in the synchronized cell populations compared to the corresponding pre-synchronized populations (Supplementary Figures S1 and Table S2). The estimation of >80% synchronization for M phase cells was based on the observation of characteristic traits described for cultures undergoing a *nuc2* arrest (26); increased septation index (from ∼16% to ∼50%), highly condensed chromosomes, and the presence of enucleate cells, following DAPI staining.

### Chromatin isolation for Genome Conformation Capture

Chromatin isolation and Genome Conformation Capture (GCC) were performed as in (5,9), with modifications; Following synchronization, cultures (200 ml) were cross-linked, washed and suspended in FA-lysis buffer. Aliquots containing ∼9.5 × 10^8^ cells were made up to a volume of 330 μl with FA-lysis buffer and the cell walls were digested with T20 Zymolyase (70 μl at 75mg/ml; 35°C, 40 min with periodic inversion) before heat inactivation (60°C, 5 min). Acid washed glass beads (500 μl) were added to each sample before disruption in a Geno/Grinder (−20°C; 1750 rpm, 2 × 30 s on 60 s off; SPEX^®^ SamplePrep 2010). Glass beads were removed by the centrifugation of chromatin through a pin hole into a clean tube (2000 rpm, 1 min). Chromatin was pelleted (13 000 rpm, 15 min, 4°C), washed with FA-lysis buffer, suspended in chromatin digestion buffer and stored (−80°C).

Each chromatin sample was aliquoted into 10 sets of 9.5 × 10^7^ cells. Samples were digested with AseI (100U, New England Biolabs, 37°C, 2 h). A ligation control (sees below and Supplementary Table S3) was added to the AseI digested chromatin, samples were diluted (∼20-fold) and ligated with T4 DNA ligase (20U, Invitrogen). Following ligation, cross-links, protein and RNA were removed. pUC19 plasmid (27.4 pg/2ml) was added as a sequence library preparation ligation control before phenol:chloroform (1:1) extraction and column purification. Three micrograms of each GCC library was sent for paired-end sequencing (50 bp, BGI China).

### Production of external ligation controls for GCC library preparation

External ligation controls were produced (5) with an AseI restriction enzyme site at one end (Supplementary Table S3) from the *Escherichia coli* genome, Lambda phage genome and pRS426 plasmid (5). The digested polymerase chain reaction products (9.5 × 10^7^ copies) were introduced into the GCC samples (i.e*. E. coli*: G1 phase, pRS426: G2 phase, Lambda: M phase) prior to the ligation step of the GCC protocol. Following sequencing, only one ligation event was detected between the pRS426 ligation control and an AseI fragment in one of the G2 phase biological replicate. A number of ligation events were also detected between the *S. pombe* genome and the pUC19 control (G1 phase: 14, G2 phase: 7, and M phase: 2), indicating that intermolecular ligation events occurred during preparation for sequencing at the BGI.

### Network assembly

GCC networks were constructed from 50 bp paired-end Illumina Genome Analyzer sequence reads using the Topography suite v1.19 (9). Topography uses the Short Oligonucleotide Alignment Program (SOAP) algorithm (27) to position Paired-End (PE) tags and single ends which contain a AseI restriction enzyme site onto the *S. pombe* (*S. pombe* genome supplementary file) reference genome, with the inclusion of the pUC19 (SYNPUC19CV), *E. coli*, pRS426 and Lambda phage ligation control sequences. No mismatches or unassigned bases (N) were allowed during positioning.

Significant interactions were defined as those that occurred at levels above the false detection rate (FDR) cut-off value (see below). Unless explicitly stated, all bioinformatics analysis was performed on significant (≥FDR cut-off), uniquely positioned, non-adjacent (only interactions between restriction fragments that were not adjacent to each other in the linear sequence) interactions data using in house Perl and Python scripts. Except where indicated, statistical analyses were performed using R and Venn-diagrams were drawn with the Vennerable package (28).

### FDR cut-off calculations

Random ligation events can occur during the two ligation steps in the GCC protocol: (i) the ligation of the cross-linked fragments; and (ii) linker addition during preparation for sequencing. We employed two methods for the identification of significant interactions: (i) a statistical method that calculates a FDR cut-off as in (9); and (ii) the external ligation controls during the GCC library preparation allowed us to measure the rates of intermolecular ligation events. Only one intermolecular ligation event was detected and was at a frequency below the calculated FDR cut-off. Therefore, we determined our significance cut-off to be ≥3 using the statistical method described in (9).

### Collector's curve

Collector's curves are a form of rarefaction curve that plots the cumulative number of species recorded as a function of sampling effort. Here we used the collector's curve to test if we had sampled the interactions to saturation. Collector's curves were generated using the total interaction datasets (including non-significant interactions) for G1, G2 and M phases of the *S. pombe* cell cycle (5). An artificial interaction was added to each interaction dataset (e.g*.* G1, G2 or M phase) at the frequency of the calculated FDR cut-off. Increasing fractions (i.e. 10%, 20%, 30% …100%) of the modified total interaction sets were independently randomly sampled. This was repeated 100× for each modified total interaction set. For each random dataset, interactions that were sampled more frequently than the artificial interaction were considered significant (random-significant). We then plotted the percentage (average across the 100 replicates) of the significant captured interactions that were identified in the random-significant populations (Supplementary Figure S2). The collector's curves indicated that, despite the high correlation between biological replicates, the interaction network was not sampled to saturation.

### Network sampling

To ensure that the results observed were not due to an under sampled network we compared the number of significant interactions detected with those derived from randomly generated networks. The total interaction datasets (including non-significant interactions) for each cell cycle phase: G1, G2 and M, were divided into individual interactions according to the detected interaction frequency and pooled. One thousand random networks, that contained the same number of interactions as the initial input file, were generated for each cell cycle phase from both a non-reducing and reducing pool of interactions (i.e. sampling with and without replacement, respectively). The number of significant interactions in each random data file was determined using the same cut-off calculated for the real data (see FDR cut-off). Random significant interactions for the three cell cycle phases were compared to determine the number of interactions specific to each cell cycle phase and shared by different phases. A Chi-square test was then used to determine whether there was a significant difference between the expected (i.e. the average number of interactions found to be specific or shared from the 1000 random data files) and observed (i.e. the real data). The results indicate that the detected network is significantly different from a random distribution.

### RNA extraction

For RNA extraction, cells were harvested from 12 ml of each synchronized cell culture prior to cross-linking (4000 rpm, 2 min, RT), washed with 5 ml of AE buffer (50-mM Sodium Acetate, 10-mM ethylenediaminetetraacetic acid, pH 5.3) and suspended in AE buffer (400 μl). Cell suspensions were transferred to tubes containing an equal volume of phenol/chloroform/isoamyl alcohol (24/24/1 Ambion) and 400 μl of acid washed glass beads (Invitrogen). Tubes were transferred to a chilled block (−20°C) in a Geno/Grinder (SPEX sample prep 2010) and the cells were lysed (1750 rpm, 8 × 30 s on 60 s off). Lysis was completed by a freeze thaw (−80°C, ∼15 min) before centrifugation (14 500 rpm, 5 min, 4°C). The aqueous phase was extracted 3× with an equal volume of phenol/chloroform/isoamyl alcohol. RNA was isolated by precipitation with one-tenth volume ammonium acetate (5 M) and two volumes of 100% ethanol at −80°C (>30 min/overnight) before centrifugation (14 500 rpm, 10 min, 4°C). RNA was washed with 70% ethanol (350 μl; 14 500 rpm, 5 min, 4°C) and air dried (37°C, ∼15 min). RNA pellets were suspended in RNASecure (80 μl; Ambion) and dissolved by heating (60°C, 10 min). RNA concentration was determined by Nano-Drop (ACTGene ASP-3700) and 2 μg of each sample visually inspected following electrophoresis through a 1% (w/v) agarose gel. RNA was stored at −80°C before RNA sequencing (BGI China, 90-bp paired-end RNA sequencing analysis).

### Transcriptome analysis

RNA sequences (90 bp) were quality assessed using FastQC (http://www.bioinformatics.babraham.ac.uk/projects/fastqc/). To maximize the quality of the sequence reads, 10 bp was trimmed off either end of the sequences using fastx_trimmer (http://hannonlab.cshl.edu/fastx_toolkit/index.html) resulting in final sequence lengths of 70 bp.

The identification of differentially expressed genes was performed using cufflinks (29) to analyze the trimmed RNA sequences as a time course. Briefly, trimmed RNA-seq reads were aligned to the *S. pombe* reference genome (ASM294v2) using Tophat version 2.0.7 (http://tophat.cbcb.umd.edu/) without providing the *S. pombe* Gene Transfer Format (GTF) file. This allowed for novel transcript discovery. Aligned reads were assembled for differential expression analysis using cufflinks version 2.0.2 (http://cufflinks.cbcb.umd.edu/) and merged using cuffmerge (http://cufflinks.cbcb.umd.edu/manual.html#cuffmerge) with an ‘assemblies’ file containing the transcripts.gtf output files from cufflinks for the two biological replicates of each cell cycle phase in the order G1–G2–M–G1. Finally, differential expression analysis was performed using the merged.gtf output file from cuffmerge, the —T operator, and the accepted_hits.bam output files from tophat in the time series order G1–G2–M–G1.

The raw transcript levels for genes in individual biological replicates were highly correlated (*R*_2_ > 0.91). For downstream analyses, transcription datasets were divided into: (i) genomic regions that were in the top and bottom 5% of transcript levels in each cell cycle phase (Supplementary Table S4 and Supplementary File 4); and (ii) genomic regions whose transcript levels were differentially regulated during the three cell cycle transitions (G1 → G2, G2 → M, and M → G1) (Supplementary Table S5; Supplementary Files 4 and 5). Except where indicated, statistical analyses were performed in R (28). Venn-diagrams were drawn using the Vennerable package available for R.

### Gene ontology analysis

The AmiGO Term Enrichment online resource (http://amigo.geneontology.org/cgi-bin/amigo/term_enrichment) was used to determine if particular gene sets were enriched in Gene ontology terms within Pombase. We used a maximum *P*-value of 0.05 and required a minimum of two gene products for enrichment.

### Chromosome distribution of genes with high, low and differential transcript levels

The chromosomal distribution of genes with high, low and differential transcript levels was determined by calculating the central position (base pair) for each gene and using this to assign the genes along each chromosome into 50 000-bp bins (5). For the genes with high and low transcript levels the number of genes per bin was graphed (Supplementary Figure S3), while for the differentially expressed genes the average fold change in expression per bin was plotted (Supplementary Figure S4). To test whether gene sets had a non-random chromosomal distribution, one-sample Kolmogorov–Smirnov tests were performed using the genes central positions along each chromosome and significant results are displayed as *P*-values.

### Loop lengths of interactions within chromosomes

The loop length (bp) between interacting fragments was calculated for significant uniquely positioned intrachromosomal interactions detected at each cell cycle phase. Where two interacting fragments were located on the same chromosome, defined by coordinates Fn_start_ – Fn_end_; the loop length (*L*) was defined as:

\begin{equation*} {L} = |{\rm F}2_{{\rm start}} - {\rm F}1_{{\rm end}} | \end{equation*}

where interacting fragments were ordered so that F1_end_ < F2 _start_.

Loop length frequencies were plotted as histograms with bin widths of 100 or 10 000 bp.

### Determination of genome colocalization levels

We determined whether retrotransposon long terminal repeat (LTR) elements and genes with high, low and differential transcript levels were connected with themselves (colocalized) at a level different from random. The frequency of interactions that occurred between the genomic regions (i.e. colocalization) was calculated from the GCC interaction networks. Sets of random loci of the same number and length (bp) as the set of loci of interest were generated by randomly selecting a start coordinate for each locus within the set and then adding the length (bp) of the original locus of interest to obtain the end coordinate. Two populations of random sets were generated: (i) the conserved random (CR) sets conserved the number of loci per chromosome. This ensured that significant results were not due to the specific linear or spatial organization of an individual chromosome(s); and (ii) the random (R) sets where loci were randomly selected across the entire genome, with chromosome selection determined at a frequency that was relative to the chromosome lengths. One thousand random datasets were generated for the CR and R methods. The colocalization frequencies for the sets of randomly selected loci were determined in the GCC interaction networks. *P*-values were calculated as the number of times the colocalization frequencies of the original data were higher or lower than the randomly generated datasets. Colocalization frequencies were only considered significant if they were significant by both CR and R methods. The relative proportions of inter- and long versus short (<50 kb) distance intrachromosomal interactions that occurred for colocalizing regions were also determined. Colocalization figures use a per test significance level of 5%. This leads to acceptable expected FDRs ranging from 7–14% (not detected were excluded from this calculation).

### Coarse-grained polymer modeling

G1 and G2 phase chromosomes were modeled as coarse-grained flexible chromatin fibers using a Monte Carlo approach. Spatial constraints governing the nuclear size (30), and biological constraints restricting the telomere (21) and nucleolus positioning and centromere colocalization with the spindle pole body (SPB) (21,31,32) were based on microscopic observations (33). Models with the nucleolus diametrically opposite the SPB represent ≥45% of the population that has been previously observed microscopically (31). Subsets (10%) of the captured interactions for the G1 and G2 phase genomes were incorporated as attractive forces. Three model types were generated: confined, constrained and interactions. The confined model included the spatial constraints and nucleolar excluded volume. The constrained model included the spatial and biological constraints. In addition to the spatial and biological constraints, the interactions model also included captured uniquely positioned, significant, non-adjacent interactions from the GCC data. Sets of interactions for inclusion in the interactions model were randomly selected with a probability proportional to their frequency of capture in the GCC experiment. Full details for the algorithm are available in Supplementary Methods.

### Density distribution function

To calculate the DDF the set of elements of interest was mapped onto the chromosome granules. For each granule identified as containing the element of interest, the coordinates were extracted from each genome structure generated for a given set of conditions (i.e. stage of the cell cycle and restrained polymer model: confined, constrained or interactions). This allowed the evaluation of the effects of the different model constraints on the density distribution. In addition, an interactions control DDF was calculated from the interactions models using a randomly selected set containing an equal number of granules as the set of elements of interest.

Pairwise distances between mapped granules were calculated for each particular structure. The DDF value for given intervals (*R, R* + *dr*) was calculated as

\begin{equation*} DDF(R,R + dR) = \frac{{n(R,R + dR)/v(R,R + dR)}}{{N/V}} \end{equation*}

Where *n*(*R, R* + *dR*) is number of granules pairs separated by the distance (*R, R* + *dR*); *v*(*R, R* + *dR*) is volume of spherical layer within the nucleus; in the cases where the spherical layer overlapped the nuclear border the volume was corrected according to (33). *N*/*V* is the overall density of granules, where *N* is the total number of granules of interest and *V* is the volume of nucleus. DDFs were calculated for each individual structure within the ensemble of structures for each model and the mean DDF presented.

Statistical comparisons of DDFs were performed according to (33). Briefly, the area between the DDF curve and a density ratio of 1 was calculated for each condition. To exclude effects due to short range linear clustering within the chromosomes, the area between the DDF curve and a density ratio of 1 was calculated starting from the distance equal to three granule diameters (90 nm for G1 type models, 180 nm for the G2 type models). Two-tailed unpaired *t*-tests were performed to determine the significance of the difference between the means in pairwise comparisons.

### Relative LTR and gene density maps

To assess the preferred positions of LTRs and genes across the nucleus, we calculated their relative density maps. The relative density maps show the proportion of granules containing element of interest at the given point within the nucleus, averaged across an ensemble of structures.

G1 and G2 phase *S. pombe* nuclei display rotational symmetry with respect to the SPB-nucleolus axis. Thus, external restraints (i.e. centromere, telomere and nucleolus positions) and chromosome interactions are equally fulfilled if the whole genome conformation is rotated around the SPB-nucleolus axis by an arbitrary angle. Therefore, the 3D structure can be projected onto a two-dimensional (2D) map. This approach has been used for structural analyses of the *Saccharomyces cerevisiae* genome structure (33–35).

Using the approach, the positions of the elements of interest, within the 3D structure, present as an array of points in a 2D plane. Once mapped onto the 2D plane, we were able to calculate the density of these points across the nuclear space. Briefly, we mapped the array of points onto a 2D rectangular grid according to (32). The density of the elements of interest was calculated according to:

\begin{equation*} {\rm Density} = \frac{{{\rm Frequency}(z_{{\rm pixel}} ,r_{{\rm pixel}} )}}{{\pi \Delta _z \left( {(r_{{\rm pixel}} + \Delta _r )^2 - r_{{\rm pixel}}^2 } \right)}} \end{equation*}

\begin{equation*} \begin{array}{*{20}l} {{\rm Frequency}(z_{{\rm pixel}} ,r_{{\rm pixel}} )} \\ { = \frac{1}{{N_{{\rm structures}} }}\sum\limits_{{\rm Structures}} {\sum\limits_{{\rm Granules}} {\frac{1}{{2\pi \sigma ^2 }}{\rm exp}} } } \\ {\left( { - \frac{{(z_{{\rm pixel}} - z_{{\rm granule}} )^2 + (r_{{\rm pixel}} - r_{{\rm granule}} )^2 }}{{2\sigma }}} \right)} \\ \end{array} \end{equation*}

Here Δ_z and Δ_r are the grid size in the z and r directions respectively. *z*_granule_ is the projection of the granule onto the SPB-nucleolus axis; and *r*_granule_ is the radial distance between the granule and axis of symmetry; *z*_pixel_ and *r*_pixel_ are pixel grid coordinates; *σ* is the granule size; *N*_structures_ is the number of structures in the ensemble. For the G1 and G2 density plots *σ* was set to the radius of granules (i.e*.* 15 or 45 nm, respectively).

The nuclear space was represented as a 2D grid consisting of: G1 (266 × 266) pixels; and G2 (342 × 342) pixels, with a grid size equal to $\Delta _z = \Delta _r = 10\;{\rm nm}$. 2D maps were calculated for elements of interest extracted from ensemble of structures generated under each set of conditions.

To exclude the effect of general genome compaction due to the model restraints, the density of elements of interest was normalized by total chromosome density:

\begin{equation*} {\rm Relative}\;{\rm {\rm Density}}({\rm Element}) = \frac{{{\rm Density}({\rm Element})}}{{{\rm Density}({\rm total}) + Dempf}} \end{equation*}

Here ‘Density(total)’ is the density of the whole genome granule set and *Dempf* is a small adjustment, which negates a discrete noise in the zones of low absolute density—nuclear periphery. The value of *Dempf* was chosen as the density of one granule at the nuclear periphery *r* = *R*_nucleus_. In the density maps obtained in our study the majority of the nuclear area has an absolute density 5× higher than *Dempf*, so the influence of *Dempf* on the relative density can be neglected for most pixels.

## RESULTS

### The genome organization of *S. pombe* changes throughout the cell cycle

To determine how genome organization changes through the cell cycle, we used three cell division cycle (*cdc*) mutants: *cdc10–129*, *cdc25–22* and *nuc2–663* that block *S. pombe* cells in the G1, G2 and Mitotic anaphase stages of the cell cycle, respectively. We chose to use these temperature sensitive mutants for synchronization because: (i) we could obtain mutants in the same genetic background; (ii) the temperature shift was identical for all strains; (iii) these mutations are well characterized (17,26,36); (iv) a single method can be used to isolate cells in the three phases; and (v) we could obtain high levels of synchronization.

Cultures of *S. pombe* strains MY291 (*h- lue1 cdc10–129*), MY284 (*h- lue1 cdc25–220*) and MY286 (*h- lue1 nuc2–663*) were synchronized in G1, G2 and M phase by shifting to a restrictive temperature (‘Materials and Methods’ section). The levels of synchronization were >95%, >95% and >80% for the G1, G2 and M phase cultures, respectively (see Supplementary Figure S1 for representative images of the synchronized cells). The isogenic strains used in this study responded similarly to the identical, instantaneous temperature shift, but it remains possible that some of the observed differences in the captured interactions were indirect consequences of the mutations used to arrest the cell cycle. While we cannot exclude this possibility, we interpret the conservation of a core interaction set common to all three strains (see below) as evidence that allele-specific effects were rare.

Chromosomal interactions were captured by GCC (9). The chromosomal interactions that were captured for the independent biological replicates for each of the G1, G2 and M phases of the cell cycle were highly correlated (Supplementary Figure S5A–C). All subsequent analyses were performed on captured interactions in which both of the interacting fragments map to a unique position (hereafter termed ‘uniquely positioned’) within the *S. pombe* reference genome (ASM294v2), unless otherwise stated.

**Figure 1.**
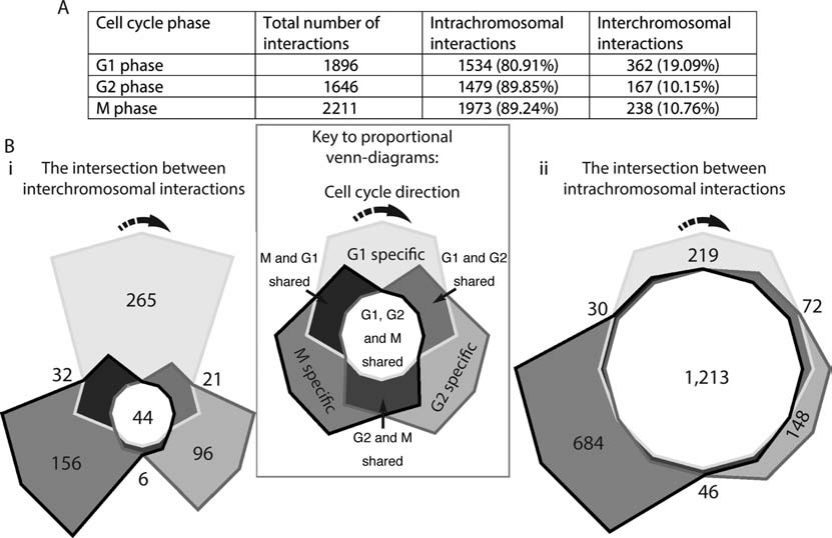
Genome interactions change throughout the *Schizosaccharomyces pombe* cell cycle. (**A**) Significant captured interactions between non-adjacent uniquely positioned restriction fragments were tabulated according to whether they were inter- or intrachromosomal (‘Materials and Methods’ section). Most interactions were intrachromosomal, especially following replication and entry into the G2 and M phases. Intersections between the (**Bi**) inter- and (**Bii**) intrachromosomal interactions captured in the three cell cycle phases were calculated and plotted as proportional Venn-diagrams in which the area reflects the number of interactions. (**Bi**) The majority of captured interchromosomal interactions were specific to one cell cycle phase. (**Bii**) By contrast, the majority of captured intrachromosomal interactions were shared among the three cell cycle phases.

Consistent with other studies (8,9,12), the majority (∼80% in G1 and ∼90% in G2 and M phases) of the captured interactions were intrachromosomal (Figure 1A). This supports the existence of chromosome territories in *S. pombe* throughout the cell cycle (8,37). Moreover, the captured interactions were highly similar between the G1 and G2 phases (Supplementary Figure S5D). By contrast, there was poor correlation between the interactions in the G2 and M or M and G1 phases (Supplementary Figure S5E and SF, respectively). This agrees with previous observations of a loss of correlation for intrachromosomal interactions between the G1 and M phases in HelaS3 cells (12).

The intersections between the captured inter- and intrachromosomal interactions from the three cell cycle phases were calculated and plotted as Venn-diagrams in which the area of each segment is proportional to the number of interactions (Figure 1B(i) and (ii), respectively). There were a total of 767 inter- and 4986 intrachromosomal interactions captured in the three stages of the cell cycle (Figure 1A). Therefore, the inter- and intrachromosomal interaction Venn-diagrams have been drawn on different scales and polygons have been used to enable the visualization of small areas which occur at the intersections of the interaction profiles.

The Venn-diagrams for the inter- and intrachromosomal interactions have dramatically different shapes. This difference in shape is predominantly due to the relative contributions of the interactions that were shared by all three cell cycle phases (compare the central segments of Figure 1B(i) and (ii)). Interactions between chromosomes were predominantly cell cycle specific, with the largest number forming in G1 phase (Figure 1B(i)). M phase chromosomes had the largest number of phase-specific intrachromosomal interactions, with a clear increase in the number of interactions with loop lengths of ≤5 kb (Supplementary Figure S6). We interpret the increased proportion of intrachromosomal:interchromosomal interactions in the G2 and M phase-specific interactions as reflecting contacts between replicated chromatids in G2 on the one hand, and further compaction of the mitotic anaphase chromosomes on the other. Comparisons of the distributions of captured interactions with randomly generated interaction sets, prepared using both reducing and non-reducing sampling (‘Materials and Methods’ section), confirmed that the differences in the intersections that we observed are not due to network under sampling (Supplementary Figure S2 and Table S6).

Two-dimensional heat maps of interactions captured between unique and repetitive sequences on each chromosome (Supplementary Figure S7) revealed preliminary evidence for TADs along each chromosome, similar to those observed in metazoans (14,15). One domain was found on chromosome II while at least two such domains were located towards the ends of chromosome III. These domains contained interactions within and between unique and repetitive elements, which are highly enriched on chromosome III. Interestingly, the TAD-like domains on chromosome III were adjacent to the rDNA repeats on the left and right arms of chromosome III. Being located next to the largest repeat arrays in the *S. pombe* genome may help explain the observation that these chromosome III TAD-like domains did not disappear during M phase unlike human TADs (12).

### Long terminal repeats contribute to cell cycle-specific genome organization and gene expression

In contrast to intrachromosomal interactions, interchromosomal interactions reflect clustering of elements from different chromosomes. For example, the colocalization of telomeres and centromeres is well known from microscopic studies in *S. pombe* (38). Consistent with this, we detected inter- and intrachromosomal colocalization between the two sub-telomeric domains on Chromosomes I and II (Supplementary Figure S7). Analysis of the distances between intrachromosomal interactions revealed that these ‘full length’ interactions were enriched, indicating that all chromosomes effectively circularize into a Rabl conformation (Supplementary Figure S8). Centromeres also colocalize throughout the cell cycle (Supplementary Figure S7), consistent with previous observations that fission yeast chromosomes assume a Rabl conformation (8,22,39).

**Figure 2.**
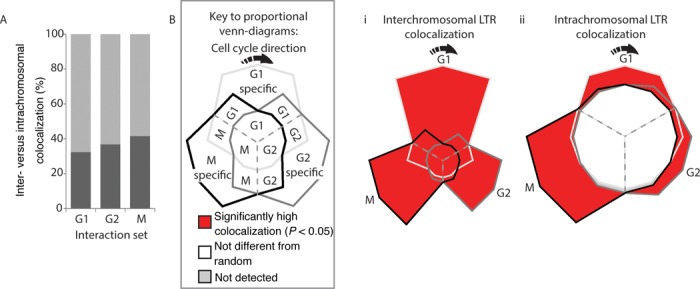
LTR colocalization predominantly occurs between chromosomes and varies thoughout the cell cycle. (**A**) The colocalization between LTRs captured in the interactions for each cell cycle phase was determined and the proportion of inter- (light gray) compared to intrachromosomal (dark gray) LTR colocalization was calculated. The majority of LTR colocalization occurs between chromosomes rather than within chromosomes. (**Bi**) The frequency with which LTR elements from different chromosomes colocalize in the different interchromosomal subsets (Figure 1Bi) was compared to randomly selected genomic regions (see ‘Materials and Methods’ section). LTRs from different chromosomes colocalize at a significantly high frequency in all three phases of the cell cycle. (**Bii**) Intrachromosomal LTR colocalization also occurred at a significantly high frequency in G1, G2 and M phases, but was not different from random among interactions that were shared by all three cell cycle phases (center) and was not detected in the G2–M phase shared interactions. Individual *P*-values are presented in Supplementary Table S7. The expected false detection rate (FDR) for B(i) and (ii) is 7%.

LTRs are the predominant family of repetitive elements found within the *S. pombe* genome, and are highly enriched on chromosome III. They are bound by the CENP-B protein Abp1 and colocalize into *Tf* bodies (40,41). However, it is unknown if the spatial environment in which LTR elements reside changes throughout the cell cycle. Analyses of uniquely positioned interactions that overlapped LTR boundaries identified LTR colocalization within our dataset consistent with previous observations in *S. pombe* (8,40–42). Colocalization between LTRs occurred predominantly between LTRs located on separate chromosomes (Figure 2A). Moreover, G1:97%, G2:100% and M:94.1% of the observed intrachromosomal LTR colocalization involved interactions that were between two LTRs >50 kb apart. This is consistent with ‘regulated’ LTR colocalization rather than accidental associations due to general chromosome compaction.

We determined the frequency with which LTR elements colocalized with each other in the interaction subsets and superimposed this onto the interaction Venn-diagrams from Figure 1. Significantly high levels of LTR colocalization were detected for all interchromosomal interaction subsets (Figure 2B(i)). Similarly, significantly high levels of LTR colocalization were observed within the captured intrachromosomal interactions except for the G2-M and G1-G2-M shared interaction sets (Figure 2B(ii)). The finding that LTR colocalization was enriched within the specific interaction sets is consistent with different subsets of LTR–LTR interactions forming throughout the cell cycle.

A strong intrachromosomal interaction was detected between two restriction fragments on chromosome II (259 508 bp apart) that contain LTR elements (Supplementary Figure S8 and Table S8). This interaction occurred at a very high frequency in G1 phase, disappeared in G2 phase and then returned with the highest frequency in M phase (Supplementary Figure S8). Analyses of transcripts present at each cell cycle phase (‘Materials and Methods’ section) revealed that the disappearance of the LTR interaction in G2 phase correlated with the transcriptional upregulation of an ubiquitin-protein ligase gene (SPBC21D10.09c) that overlapped one of the interacting fragments (Supplementary Tables S8 and S9). This is consistent with earlier observations that the disruption of LTR colocalization results in the upregulation of nearby genes (40,41).

### LTR containing genomic regions have preferred positions within the *S. pombe* nuclear space

To further investigate the spatial organization of *S. pombe* chromosomes within the nucleus we developed coarse-grained polymer models of the genome (Supplementary Methods). Three different types of models were used for the G1 and G2 phases of the *S. pombe* genome: (i) confined; (ii) constrained; and (iii) interactions model (Supplementary Methods). The confined model is a control in which only the nuclear shape and volume, including exclusion of the chromosomes from the nucleolus, is imposed on the simulated chromosome polymers. In addition, the constrained and interactions models included biological restraints on the chromosomes. Specifically, in the constrained model the chromosomes are subject to tethering restraints (i.e*.* centromere and telomere positioning) but are randomly configured within the confinement volume. In the interactions model the chromosomes are subjected to the tethering restraints and randomly selected subsets of the experimentally defined inter- and intrachromosomal interactions (Supplementary Methods). Experimentally defined interactions were chosen randomly for incorporation into the interactions model because it is not technically possible to isolate the total set of interactions that co-exist within a single synchronized cell from the GCC datasets (33).

**Figure 3.**
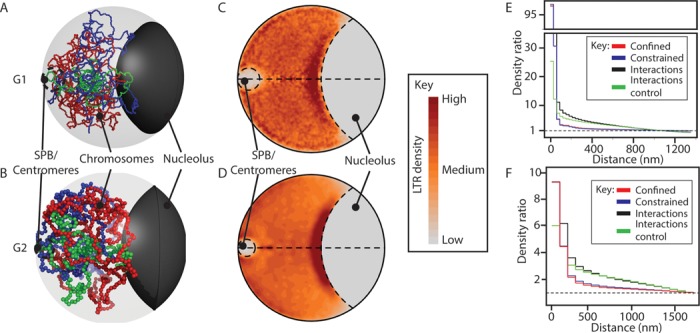
Polymer models indicate LTRs colocalize close to the nucleolus. (**A** and **B**) Cartoons illustrating *Schizosaccharomyces pombe* genome organization during the: (A) G1; and (B) G2 phases of the cell cycle. Ensembles of coarse-grained polymer models that incorporated biological constraints and captured interactions were generated (Supplementary Methods). Representative models are shown for each cell cycle phase. SPB refers to the spindle pole body. Chromosome I, red; Chromosome II, blue; Chromosome III, green. (**C** and **D**) Two-dimensional (2D) projections of LTR density across the ensembles of polymer models show enrichment of LTRs in a nucleolar proximal region during the: (C) G1; and (D) G2 phases of the cell cycle. (**E** and **F**) Analyses of the density distribution functions (DDFs) within the: (E) G1; and (F) G2 phases of the cell cycle. Comparisons of the DDFs determined from LTR interactions in ‘constrained’ (tethering restraints only, blue line) or ‘confined’ (spatial confinement only, red line) models indicate significant colocalization (*P* <0.001; two-tailed, unpaired *t*-test, see Supplementary Methods). This colocalization is not due to a general increase in polymer compaction as colocalization of the LTR elements in the model (‘interactions’, black line) is significantly different (*P* < 0.001) to that for an equivalent set of random loci (‘interactions control’, green line).

Since the GCC methodology and genome structures are probabilistic, we generated 500 individual structures for each type of model (see Figure 3A and B for representative structures). The relative density of the LTR elements throughout the *S. pombe* nucleus was calculated for the G1 and G2 phase interactions models and plotted as relative density maps (Figure 3C and D). These relative density maps show strong enrichment of LTRs within a region proximal to the nucleolus, consistent with preferred positioning of these elements within the nuclear space. There is also a low level of LTR enrichment around the centromeres in the G2 phase consistent with observations that LTRs are associated with centromeres (42).

We used the density distribution function (DDF), which reports on the degree of spatial clustering without making any assumptions about the size, shape or numbers of clusters (33), to measure the relative contributions of the different physical and biological restraints to the LTR clustering we observed within our models. Uniformly arranged points have a DDF of 1, while clustering is identified by values >1. The DDFs for each of the 500 structures generated with a given type of model for the G1 or G2 cell cycle phase were calculated separately and then averaged for the entire ensemble (i.e*.* all 500 structures). The DDF plots for the interactions model ensembles confirm that LTR colocalization occurs within G1 and G2 phase nuclei (Figure 3E and F). The confined and constrained model DDFs were almost identical for the G1 phase models (Figure 3E) and overlapped to a large extent in the G2 models (Figure 3F). Therefore, the LTR clustering identified in the confined and constrained models is predominantly due to the linear order of the elements within the chromosomes (33). By contrast, the introduction of the captured interactions into the interactions model causes a significant shift in the DDF when compared to both the constrained and confined models. As such, the LTR colocalization that was observed in the interactions models was affected by the interactions and was not simply an effect of the linear ordering of the elements within the chromosomes. Furthermore, comparisons of the LTR colocalization with a randomly selected set of elements (the interactions control) demonstrated that the increased colocalization was not a result of general polymer compaction due to the introduction of the captured chromosomal interactions (compare interactions model and interactions control, Figure 3E and F).

### Genes with high transcript levels throughout the cell cycle are associated with a high proportion of interchromosomal colocalization

The formation of chromosomal contacts can be due to regulatory interactions associated with transcription (8). Therefore, we sequenced the RNA transcripts present within the same cells in which the chromosomal interactions were captured (‘Materials and Methods’ section). Genes that did not have detectable transcripts within the RNA-seq data were not included in this or subsequent analyses. We analyzed these RNA-seq data to identify the genes that had high (highest 5%), low (lowest 5%) and differential transcript levels throughout the cell cycle (Supplementary Tables S4 and S5, Supplementary File 6). Genes in the high transcript level gene set (top 5%) were largely conserved between the cell cycle phases (Supplementary Figure S9A). The genes that had the lowest (5%) associated transcript levels were largely specific to each phase of the cell cycle (Supplementary Figure S9B).

**Figure 4.**
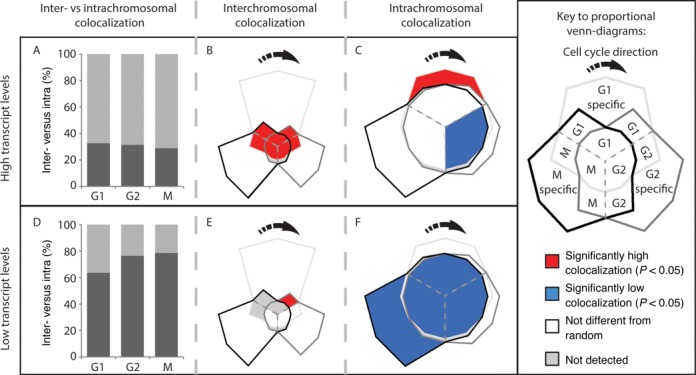
Genes with high transcript levels form cell cycle-specific interchromosomal clusters. (**A**) Inter- (light gray) and intrachromosomal (dark gray) colocalization between high transcript genes (Supplementary Table S4, Supplementary Files 4 and 6) within the captured interactions at each phase of the cell cycle was determined. Colocalization between high transcript level genes predominantly occurs between different chromosomes. (**B**) The frequency with which high transcript genes colocalize in the different interchromosomal subsets (Figure 1Bi) was compared to randomly selected genomic regions (see ‘Materials and Methods’ section). High transcript genes from different chromosomes colocalize at a significantly high frequency in the interactions shared by the M/G1, G1/G2 and G2/M transitions of the cell cycle. (**C**) Intrachromosomal high transcript gene colocalization also occurred at a significantly high frequency in the G1 phase. By contrast, highly expressed genes colocalized at a significantly low frequency not colocalized in the G1/G2 and G2/M transitions during the G2 phase in interactions that where captured at all cell cycle phases (central blue segment). The high transcript gene set is highly conserved between cell cycle phases (Supplementary Figure S9). (**D**) The proportion of intrachromosomal colocalization detected between low transcript level genes (Supplementary Table S4, Supplementary Files 4 and 6) was significantly higher than for high transcript genes (*P*-value = <0.05, R: prop.test) resulting in predominant intrachromosomal colocalization. Genes that had no detectable transcripts were excluded from this analysis. Colors as in (A). (**E**) Significant interchromosomal colocalization of low expressed genes was only observed in the G1 phase via interactions that were shared by G1 and G2. (**F**) Despite being responsible for >60% of the observed low transcript colocalization, intrachromosomal colocalization between low transcript genes occurred at or below levels expected at random. Individual *P*-values are presented in Supplementary Table S11. The expected FDR for (B, C, D and E) is 14%.

We determined the frequency with which the genes we identified colocalized in the interactions subsets. Over 70% of the colocalization of genes with high transcript levels was interchromosomal, (Figure 4A) while the intrachromosomal colocalization that occurred was predominantly between genes >50 kb apart (Figure 4A and Supplementary Table S10). Colocalization of the high transcript level genes occurred at significantly high levels in interchromosomal interactions that were shared with the G1 phase of the cell cycle (Figure 4B) and G1-specific intrachromosomal interactions (Figure 4C).

Genes within the low transcript level sets were significantly (G1, *P* < 0.05; G2, *P* < 0.01; M, *P* < 0.01) more likely to colocalize intrachromosomally than genes with high transcript levels (compare Figure 4A and D). Furthermore, in comparison to high transcript genes, there is a clear reduction in the intrachromosomal colocalization of low transcript genes separated by distances of >50 kb (Supplementary Table S10). The observed increase in the proportion of low transcript gene intrachromosomal colocalization, when compared to the high transcript genes, in the G2 and M phases of the cell cycle (Figure 4D) was not mirrored by increases in the colocalization frequency (Figure 4F). Significant interchromosomal colocalization of genes with low transcript levels only occurred in interactions that were shared by the G1 and G2 phases of the cell cycle (Figure 4E). The observed shift from a significantly high level of low transcript gene colocalization to a level not different from random within the G1 and G2 shared interchromosomal interactions (Figure 4E) is due to differences in the G1 and G2 low transcript level gene sets and changes in the interaction contact frequencies (Supplementary Figure S9B). There were several instances where interchromosomal colocalization was not detected for low transcript gene sets (Figure 4E). These results are consistent with the high and low transcript level genes existing in distinct spatial environments.

### Genes that are upregulated during the G1→G2 phase transition and downregulated during the G2→M phase transition colocalize in nuclear space

**Figure 5.**
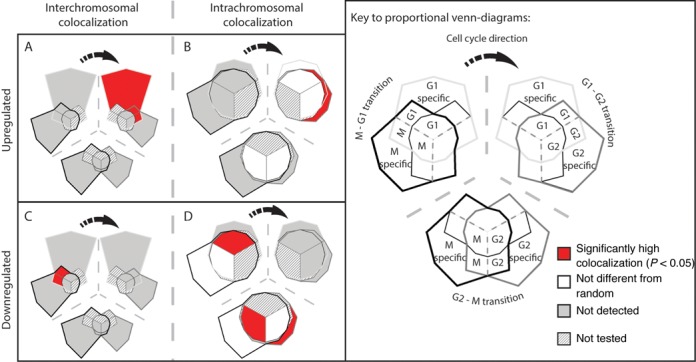
Differentially regulated genes colocalize in a cell cycle-specific manner. Genes that had a ≥2-fold change in transcript level during each cell cycle transition were identified (Supplementary File 5). We then determined whether genes with up- or downregulated transcript levels colocalized at a frequency significantly different from randomly selected genomic regions. A significantly high level of (**A**) inter- and (**B**) intrachromosomal colocalization was detected between genes that were upregulated during the G1→G2 transition. (**C**) Interchromosomal colocalization of downregulated genes occurred at a significantly high level in captured interactions that were shared by M and G1 phases of the cell cycle. (**D**) Intrachromosomal colocalization of downregulated genes occurred at a significantly high level in the M→G1 and G2→M phase transitions. Venn-diagrams were modified to remove the interaction subsets that were not tested during specific cell cycle phase transitions. Individual *P*-values are presented in Supplementary Table S12. The expected FDR for (A–D) is 10%.

Alterations in gene transcription have been associated with changes in the 3D position of a gene(s) (43,44) and the formation or breakage of DNA contacts (8). As for the high and low transcript level genes (Figure 4), we determined the colocalization frequency between genes that showed a ≥2-fold change in transcript level during cell cycle transitions (Figure 5, Supplementary Table S5 and Supplementary File 5). There was no detectable colocalization for many of the differentially regulated gene sets (Figure 5). However, genes that were upregulated during the G1→G2 cell cycle transition had high levels of interchromosomal colocalization in interactions specific to the G1 phase and shared between the G1 and G2 cell cycle phases (Figure 5A). These genes were also highly colocalized in intrachromosomal interaction subsets shared between G1 and G2, and in the subset specific to G2 (Figure 5B). Comparisons of the proportions of inter- and intrachromosomal colocalization for the differentially expressed (Supplementary Figure S10), high and low transcript gene sets revealed a reduction in intrachromosomal colocalization of differentially expressed genes involving elements separated by >50 kb (Supplementary Table S10). This was especially noticeable for genes that were upregulated on entry into M phase or downregulated upon entry into the M or G1 phases (Supplementary Table S10). Thus the genes that are differentially regulated as they enter and exit the M phase are typically involved in short range intrachromosomal connections that are less likely to be affected by M phase chromosome condensation.

Genes that were downregulated during the G2→M phase transition were significantly colocalized in the G2 phase-specific intrachromosomal interactions (Figure 5D), but were not detected to colocalize in the interchromosomal interactions (Figure 5C). There was significantly high intrachromosomal colocalization of downregulated genes in the M→G1 and G2→M phase transitions inferred from the shared interactions among all conditions (central segments, Figure 5D). This is consistent with clustering contributing to the downregulation of these genes. Collectively, these results indicate that colocalization is not required for the co-regulation of genes but may facilitate it during specific cell cycle phases.

### Genes have preferred positions in nuclear space

Our analyses of the captured interactions did not identify an obvious rule that explains how the differentially expressed genes colocalize with each other in space. This may result from the fact that proximity-based ligation methods, including GCC, require that two loci are physically cross-linkable in order to be captured. However, spatial clustering within the nuclear space does not require that loci are physically connected and thus they may remain undetectable by proximity ligation. Therefore, we tested our ensembles of coarse-grained polymer models of the *S. pombe* G1 and G2 phase chromosome organization to confirm our observations of gene colocalization within the nucleus. Genes with high, low or differential transcript levels were mapped onto the coarse-grained polymer models (Supplementary Methods). The relative density of the gene sets was averaged over each ensemble of models and plotted as a gene density map projected into two dimensions (Supplementary Methods).

**Figure 6.**
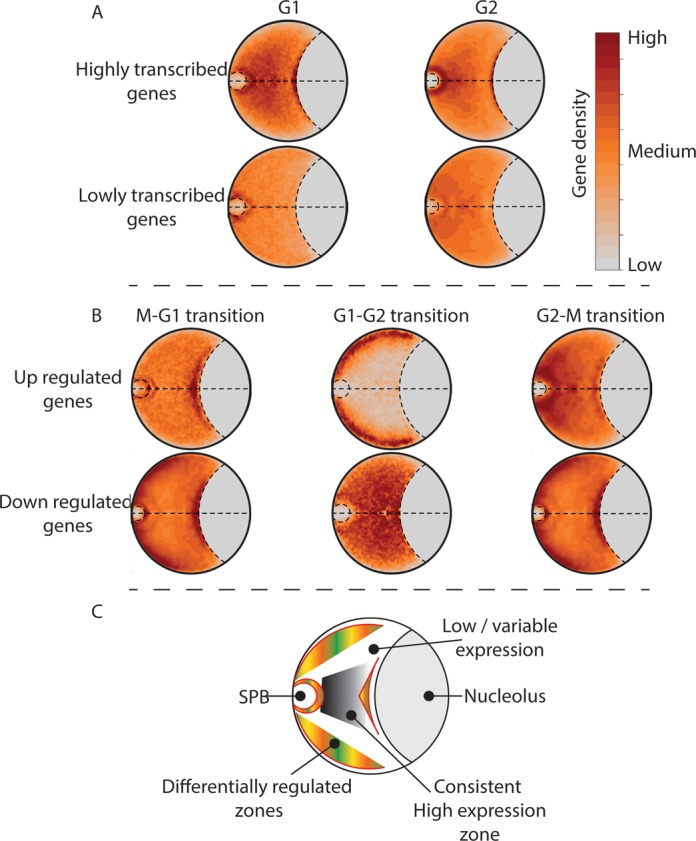
Highly transcribed and differentially regulated genes have preferred positions in *Schizosaccharomyces pombe* nuclei. Genes with high, low or differential transcript levels were mapped onto coarse-grained polymer models. The relative densities of the gene sets were averaged over all model ensembles and plotted as 2D gene density maps (Supplementary Methods). Here we present the gene density maps for the interactions models (see Supplementary Figure S11 for gene density maps of the confined and constrained models). The positions of genes that had differential transcript levels during the G1→G2 transition were mapped onto G1 phase polymer models (see Supplementary Figure S12 for the G1→G2 transition gene density mapped on the G2 polymer model). (**A**) Genes with high transcript levels occupy a central region within the *S. pombe* nuclei during G1 and G2. By contrast, genes with low transcript levels are dispersed throughout the nucleus. (**B**) Genes that are upregulated during the M→G1, G1→G2 and G2→M phase transitions occupy distinct nuclear sub-domains. (**C**) Cartoon highlighting the gross organization of the genes that are predominantly differentially regulated, highly and lowly expressed within the *S. pombe* nucleus during the G1 and G2 cell cycle phases. SPB: spindle pole body.

In agreement with earlier studies (33–35), the inclusion of tethering restraints into the polymer models (Supplementary Methods, Model restraints) contributed to the organization of the chromosomes within the constrained models when compared to the confined models (compare Figure 6 and Supplementary Figure S11). However, when the randomly selected subsets of inter- and intrachromosomal interactions were included in the interactions models, the variability within the ensemble was further reduced and made a significant contribution (*P* < 0.001) to the observed colocalization of genes (Supplementary Figure S13 and S14), including genes that were not involved in the specific interactions that were added to the model.

Genes that exhibit high transcript levels exhibit a propensity to be localized toward the center of the G1 and G2 phase nuclei (Figure 6A) while lowly transcribed genes are diffusely distributed across the nuclei with some enrichment around the SPB (Figure 6A). In contrast to both the highly and lowly transcribed genes, differentially regulated genes exhibit localized distributions that differ according to the cell-cycle phase transition (Figure 6B and Supplementary Figure S11). For example, genes that were upregulated during the G1→G2 transition are located within a zone at the nuclear periphery (Figure 6B) and are enriched for ontology groups related to cell growth (Supplementary File 7). By contrast, genes that are downregulated during this transition show a more diffuse internal distribution (Figure 6B) and are enriched for ontology groups related to rRNA and ncRNA processing and translation (Supplementary File 7). Interestingly, the linear distributions of the gene sets that were downregulated during G1→G2 transition and upregulated during the G2→M transition are not significantly different from random (Supplementary Figures S4). By contrast, the gene sets that showed significant non-random linear distributions (e.g*.* upregulated during the G1→G2 transition and downregulated during the G2→M transition Supplementary Figures S4) showed strong spatial clustering within the polymer models (Figure 6 and Supplementary Figures S13 and S14). Collectively, these results are consistent with genes in the *S. pombe* nucleus being organized within five sub-nuclear domains according to their transcriptional activity (Figure 6C). These domains are the: (i) SPB associated; (ii) nucleolus; (iii) nucleolar proximal; (iv) peripheral; and (v) central domains (Figure 6C).

## DISCUSSION

In this study, we performed the first high-resolution analyses of the structure of the *S. pombe* genome through the G1, G2 and M phases of the cell cycle. We clearly identified spatially defined nuclear sub-domains within which there is a preference for gene colocalization associated with gene regulation during cell cycle transitions. Known hallmarks of fission yeast genome organization, such as the colocalization of centromeres and telomeres (22,45) were present at each phase of the cell cycle. Moreover, mixtures of stable and dynamic interactions were detected within and between chromosomes including when the chromosomes were captured in the M phase. These results lead us to propose a refined concept of the spatial organization of the *S. pombe* nucleus in the G1 and G2 phases.

The structure of isolated metaphase chromosomes has been extensively investigated (46–48). Metaphase chromosomes are thought to contain a continuous chromatin network that is constrained by isolated chromatin-crosslinking elements spaced by ∼15 kb (48). The abundance of loops with a length ≤5 kb in M phase fission yeast chromosomes is *in vivo* evidence for the formation of a continuous chromatin network. However, the absence of a predominant loop length suggests that there is no regular coiling of the chromatin fibre. Instead our results are consistent with the chromosomes assuming dynamic ‘polymer melt’ like structures as observed for higher eukaryotes (12,49).

Early observations of connections between metaphase chromosomes (50,51) were thought to be an artefact of chromosome isolation (52). Later work demonstrated that these interactions occurred and were DNA based (53,54). Our results confirm these findings and provide the first evidence for DNA based connections between chromosomes during the mitotic anaphase within lower eukaryotes. Moreover, our finding that there is a high level of mitotic anaphase interchromosomal colocalization between LTR elements, which are bound by the CENP-B homologue Abp1 (40,41), implicates repeat regions as participating in these M-phase interchromosomal linkages. This is consistent with the finding that satellite DNA was involved in connections between mitotic chromosomes in mouse cell lines and that CENP-B was a component of the thread (54). It remains possible that the connections between the M phase chromosomes we observed resulted from contamination by unsynchronized cells. We contend that contamination was not a significant contributor to these interactions based on the fact that Naumova *et al.* also observed interchromosomal interactions between HeLaS3 M phase chromosomes (12). However, Naumova *et al.* concentrated their analysis on intrachromosomal M phase interactions because their analysis was complicated by the abnormal karyotype of these cells (12). Furthermore, our observation that genes with consistently high transcript levels exhibited significantly high colocalization in interactions that formed in the M phase and were maintained in the G1 phase is consistent with bookmarking facilitating post-mitotic reactivation (55–57). Thus, we propose that the interchromosomal interactions that occur during the eukaryotic M phase help position the chromosomes and contribute to transcriptional memory, upon entry into G1 phase.

Transcriptional silencing of LTR elements and associated genes is achieved by the recruitment of class I and II histone deacetylases to these elements and their association with *Tf* bodies (40,41). We interpret the high level of colocalization between LTR elements on different chromosomes, and at long distances within chromosomes (i.e*.* >50 kb), as further evidence for such bodies. The correlation between LTR interaction and transcript levels for the ubiquitin-protein ligase gene (SPBC21D10.09c) supports a role for LTRs in the regulation of transcription at a distance. While this is only one example, further support for LTRs having a global role in transcription regulation is inferred from: (i) the finding that LTRs exhibit cell cycle phase-specific colocalization with each other; and (ii) the preferential positioning of LTRs at the nucleolar periphery in a region that also exhibited variable gene regulation (compare Figures 3 and 6B).

Highly expressed genes have been shown to preferentially colocalize in fission yeast (8). These findings are often interpreted as indicating that transcription and/or transcription factories are involved in the spatial organization of genomes (58). The high level of genome connectivity between constitutively highly transcribed genes, in all stages of the cell cycle, suggests an extended association with transcription factories when compared to individual, cell cycle-specific genes. Moreover, the observation that interactions between genes that were highly transcribed in the G1 phase are maintained through S phase into G2: (i) indicates that interactions are either re-established or not broken during chromosome replication; and (ii) implicates these interactions in the maintenance of transcript levels. Of course, other consequences of high level transcription during S phase, such as replication stalling and DNA repair, might also contribute to colocalization (59).

Colocalization between genes is not a requirement for their co-regulation (60), but may contribute to it in specific situations (61). Our data clearly shows that genes that had high transcript levels throughout the cell cycle were highly connected and non-randomly distributed within the chromosome sequences and nuclear space. By contrast, genes with low transcript levels were lowly connected, predominantly cell cycle phase specific, and did not cluster within the chromosome sequences or within a defined spatial region of the *S. pombe* nucleus. Critically, two subtelomeric regions gene sets (Supplementary File 8) that have been previously shown to contain cell-cycle regulated genes (62) showed distinct spatial positioning within the *S. pombe* genome polymer models (Figure 6B). Collectively, our results and those published previously (33–35,63) clearly link the position of a gene within the linear sequence with its spatial position. This is consistent with the hypothesis that the observed linear clustering of specific gene sets is under evolutionary selection for increased spatial colocalization and thus more efficient co-regulation.

The re-location of genes toward or away from the nuclear periphery has been linked to variable transcription in yeast (64–66) and mammalian cells (44). Furthermore, transcriptional memories and the rapid re-initiation of transcription are associated with gene positioning at the *S. cerevisiae* nuclear periphery. The sub-nuclear gene organization that we observed within the *S. pombe* genome can potentially be explained in terms of the movement of transcription factors and signaling molecules into and throughout the nucleus. The clustering of co-regulated highly and differentially transcribed genes (Figure 6) limits the requirements for transcription factor diffusion through the nucleus effectively concentrating the factors and stabilizing gene regulation. Thus, rapid and reliable control of gene expression levels can be achieved by targeted changes to transcription factors.

The conservation of interactions between the right and left telomeres on chromosomes I and II, and the regions immediately adjacent to the ribosomal repeat regions on chromosome III, means that the chromosomes are effectively circular in all cell cycle phases. This circular organization may contribute to the stabilization of chromosome ends. Moreover, our findings are consistent with polymer models that demonstrate circular chromosome structures are requirements for de-condensation of chromosomes following mitosis and the formation of chromosome territories, within biologically relevant timeframes (67,68).

## CONCLUSION

Our results demonstrate the existence of cell cycle-specific chromosome interactions within the *S. pombe* genome. The dynamic nature of these interactions, and the observed correlation with transcript levels, indicates that the interactions are regulated. The observation that subsets of these interactions are maintained even when chromosomes are fully condensed implicates genome organization in epigenetic inheritance and bookmarking. Our results suggest that: (i) structural interactions shift in response to, or to allow, cell cycle progression; and (ii) there are separate populations of structural and regulatory interactions that participate in the formation and persistence of cell cycle-specific chromatin and gene regulation. How these interactions are inherited through mitosis remains to be determined.

## ACCESSION NUMBERS

All sequencing data and processed GCC and transcriptome files are available on Gene Expression Omnibus (GEO) accession number GSE52287.

## SUPPLEMENTARY DATA

Supplementary Data are available at NAR Online.

SUPPLEMENTARY DATA
