# Supplementary material for: Chromosome conformation maps in fission yeast reveal cell cycle dependent sub nuclear structure
Source: Nucleic Acids Res. 2014 Oct 23;42(20):12585–99. doi: 10.1093/nar/gku965 (PMC4227791; doi:10.1093/nar/gku965)
Supplement: SUPPLEMENTARY DATA [file supp_42_20_12585__index.html]

Chromosome conformation maps in fission yeast reveal cell cycle dependent sub nuclear structure — SUPPLEMENTARY DATA 

# Chromosome conformation maps in fission yeast reveal cell cycle dependent sub nuclear structure

## SUPPLEMENTARY DATA

**Files in this Data Supplement:**

- SUPPLEMENTARY DATA
- SUPPLEMENTARY DATA
