# Supplementary material for: Chromosome conformation maps in fission yeast reveal cell cycle dependent sub nuclear structure
Source: Nucleic Acids Res. 2014 Oct 23;42(20):12585–99. doi: 10.1093/nar/gku965 (PMC4227791; doi:10.1093/nar/gku965)
Supplement: SUPPLEMENTARY DATA [file supp_gku965_nar-01186-n-2014-File008.docx]

# Supplementary methods

**Contents**

Supplementary methods 1

General model description 4

Monomer size 4

Model restraints 5

1) Essential restraints 5

1a) Nuclear confinement 5

1b) Chromatin excluded volume 6

2) “Flexible” restraints 6

2a) Centromere localization 6

2b) Telomere localization 7

2c) Nucleolus localization 7

2d) Chromatin persistence length 8

2e) GCC-derived chromosome interactions 8

Scoring function 9

Monte-Carlo approach for structure optimization 9

Selection of harmonic potential strength constants 9

Chain displacement algorithm 10

Type I displacement. 11

Type II displacement. 11

Model generation procedure 11

Step 1: Structure initialization 11

Step 2: Compress chromosomes within the nuclear confinement 11

Step 3: Turn on restraints 11

Step 4: Turn on the granule excluded volume 12

Step 5: Final structure optimisation 12

Model types 12

Representation of sister chromosome cohesion in the G2 model 13

Supplementary figures 15

Figure S1. Representative images of synchronized cells. 15

Figure S2. The captured chromosomal interactions were not fully saturated. 16

Figure S3. Genes with high transcript levels were non-randomly distributed across one or more chromosomes at each stage of the cell cycle. 17

Figure S4. Genes that were upregulated during the G1 – G2 transition and downregulated during the G2 – M transition had a highly significant non-random chromosomal distribution on chromosomes I and II. 19

Figure S6. There was a marked increase in the number of intrachromosome interactions with a loop length of ≤5 Kb that were captured in M phase. 22

Figure S8. The terminal sequences on the opposite arms of each chromosome colocalized to circularize each chromosome throughout the cell cycle. 24

Figure S9. Genes with high transcript levels in each cell cycle phase are highly conserved thoughout the cell cycle while genes with low transcript levels are predomenantly cell cycle phase specific. 25

Figure S10. The proportion of inter- versus intrachromosomal colocalization varied between genes with differential transcript levels. 26

Figure S11. Relative gene density maps reveal that the inclusion of tethering constraints (*i.e.* centromere association with SPB and telomere positioning at the nuclear periphery) is sufficient to provide a degree of order to sub-nuclear domains in G1 and G2 *S. pombe* nuclei. 27

Figure S12. Relative density maps for genes that are differentially regulated during the G1→G2 transition mapped onto the ensemble of structures generated using the interactions model for the G2 phase of the cell cycle. 28

Figure S13. Genes that are highly transcribed in G1 or upregulated during the M-G1 phase transition exhibit significant inter-gene clustering that is not explained by linear chromosome structure or general genome compaction. 29

Figure S14. Genes that are upregulated during the G1-G2 transition exhibit significant inter-gene clustering within the G2 phase models that is not explained by linear chromosome structure or general genome compaction. 30

Supplementary tables 31

Table S1. *Schizosaccharomyces pombe* strains used in this study. 31

Table S2. The synchronization efficiency for each of the G1 and G2 cell cycle phase biological replicates was calculated by comparing the proportion of cells with a septum before and after synchronization. 31

Table S3. Ligation controls used in this study. Three short DNA sequences were amplified from the *E. coli* genome, pRS426 plasmid, and Lambda phage DNA. 31

Table S4. Table highlighting the number of chromosomal loci that had the highest (top 5%) and lowest (bottom 5%) transcript levels at each cell cycle phase and whether they were differentially regulated during cell cycle transitions. 32

Table S5. The number of genes that were significantly differentially regulated during each *S. pombe* cell cycle transition. 32

Table S6. The sampled network is significantly different from that expected for randomly selected interactions. 33

Table S7, p values for significant LTR colocalization sets for Figure 2. 33

Table S8. Chromosomal coordinates and lengths of the restriction fragments involved in a high frequency intrachromosomal interaction detected within chromosome II (Supplementary Figure S4). 33

Table S9. The absence of the interaction between two LTR elements in chromosome II during G2 phase was associated with the up regulation of the overlapping ubiquitin-protein ligase E3 gene. 34

Table S10. There was a reduction in intrachromosomal colocalization of differentially regulated genes that were >50 Kb apart. 35

Table S11, p values for significant colocalization of genes with high and low transcript levels for Figure 4. 35

Table S12, p values for significant colocalization sets for Figure 5. 36

# General model description

G1 and G2 phase chromosomes were modelled as coarse-grained flexible chromatin chains using a Monte Carlo optimization technique in which trial conformations are generated by a specially developed chain displacement algorithm, and accepted or rejected according to whether they fulfil the model restraints. In addition to the physical nature of the polymer (thickness, modelled by excluded volume, and persistence length), tethering restraints (Figure 1) were defined based on microscopic observations: nuclear size (1); telomere clustering (2), nucleolus positioning, and centromere co-localization with the spindle pole body (SPB) (2–4). Finally, subsets of the GCC-captured interactions were incorporated as attractive forces and imposed on the model. Sets of interactions for inclusion were randomly chosen, incorporating a bias reflecting the frequency of detection in the GCC data.


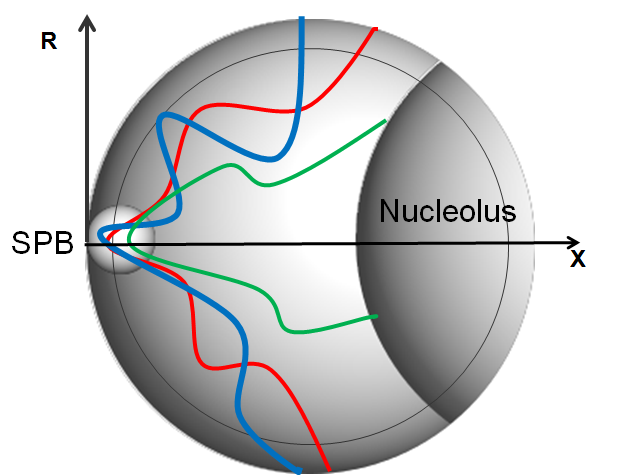


Figure 1. Schematic diagram of the generalized layout of the *S. pombe* nucleus within polymer models of the G1 and G2 phases of the cell cycle.

The coarse-grained genome models that were generated included restraints that: 1) limited the volume to the nuclear space (“confined” model); 2) positioned the nucleolus opposite the SPB; 3) promoted the colocalization of centromeres and the SPB; and 4) promoted the positioning of the telomeres within the peripheral 100nm layer of the nucleus (tethering restraints, “constrained” model). Chromosome I, red; chromosome II, blue; chromosome III, green.

# Monomer size

The chromosomes were modelled as chains of spherical chromatin granules, or monomers. The size of the monomers in G1 phase was estimated according to the chromosome compaction ratio of eukaryotic DNA. This has been measured as 7–10 nucleosomes per 11nm in *S. cerevisiae* (5); we assumed it to be similar for *S. pombe* chromosomes. We therefore represented each G1 phase *S. pombe* chromosome as a chain of connected spheres of 30nm diameter, each representing 3.5 Kb of DNA, so that the 14 Mb G1 phase genome was represented by 3583 granules.

The number of *S. pombe* chromosomes is doubled in the G2 phase of the cell cycle following DNA replication. However, the chromosome copies remain linked together by cohesin complexes (6–8). Thus, we reasoned that two copies of one chromosome may be replaced by one thicker chromosome. This hypothesis was tested using Chip-seq cohesin data from synchronized *S. pombe* cells (9) to hold chromosome copies together as described below in the section *Representation of sister chromosome cohesion in the G2 model*. As a result of these tests, the G2 phase chromosomes are described as a single thick chromosome, comprising a chain of connected spheres of 90 nm diameter. In total, the *S. pombe* G2 genome is modelled by 1195 granules, each containing 10.5 Kb DNA.

# Model restraints

Two types of restraints were incorporated into the coarse-grained genome models. Similar types of restraints were used in *S. cerevisiae* genome models(10–12). In *S. pombe* G1 and G2 models, these restraints were divided into two categories: 1) Essential; and 2) Flexible (Table 1).

**Table 1**.Restraints used in the coarse-grained polymer models. The restraints were divided into two categories: “essential” restraints must be achieved in every structure; “flexible” restraints need not be satisfied, but an energy penalty is incurred if they are not.

| **Restraint type** | **Restraints** |
| --- | --- |
| 1) Essential | a) Chromatin chain excluded volume  b) Nuclear confinement |
| 2) Flexible | a) Chromatin persistence length  b) Centromere localization  c) Telomere localization  d) Nucleolus excluded volume  e) GCC derived chromosome interactions |

# 1) Essential restraints

Essential restraints characterized nuclear properties that must be achieved in every structure. Any trial chromosome structure that violates these restraints is rejected and the simulation returns to the previous structure. There are two essential restraints present within the model: a) nuclear confinement; and b) chromatin excluded volume.

## 1a) Nuclear confinement

The nucleus was defined as an impenetrable sphere, centred at (*0,0,0*). The lengths and radii of the cells at different stages of the cell cycle (Table 2) were estimated from imaging experiments (1). From these cell dimensions, the cell volumes were calculated by approximating cell shape as a cylinder with joined half- spheres caps, such that:

$$V_{cell}\text{= 3/4 π}R^{3}\text{+π}\text{L}R^{2};$$

where $R$ is the radius of the cylinder and spherical caps and$L$is the length of the cylinder.

As the ratio of the nuclear to cell volume is 0.089 ± 0.017 (coefficient of correlation *r*=0.97, *n*=2136) and remains almost constant throughout the *S. pombe* cell cycle (1), the nuclear volumes in the G1 and G2 cell cycle phases can be estimated from the corresponding cell volumes according to:

${V_{nucleus}=0.089 V}_{cell}$;

The radius of the nucleus was calculated as 1.3 $\mu m$ and 1.7 $\mu m$ for the G1 and G2 phase synchronized cells, respectively (Table2), assuming that the nucleus is spherical.

**Table 2**. Cell and nuclear volumes, and nuclear radii for G1 and G2 phase models calculated from the mean experimentally determined cell length and diameter in each stage of the cell cycle.

| **Cell cycle stage** | **Mutant name** | **Mean cell length (μm)** | **Mean cell diameter (μm)** | **Cell volume (μm^3^)** | **Nuclear volume (μm^3^)** | **Nuclear radius (μm)** |
| --- | --- | --- | --- | --- | --- | --- |
| G1 | cdc10-129 | 8 | 5 | 124.35 | 9.95 | 1.33 |
| G2 | cdc25-22 | 15 | 5 | 261.79 | 20.94 | 1.71 |

## 1b) Chromatin excluded volume

To simulate the excluded volume of the chromatin, the solid spherical granules making up each chromosome are forbidden to overlap. Any trial conformation with overlapping granules is rejected. Granule overlaps were checked by calculating the granules pair-distance using the “Cell linked list algorithm” (13).

# 2) “Flexible” restraints

The restraints classified as “flexible” are those obtained from imaging (2–4) (*i.e.* the tethering restraints) and chromosome interaction experiments (this study). These restraints were incorporated into the model as harmonic potential energy functions. The sum of the restraint potential energy terms defines the scoring function, a measure of how consistent the structure is with the imposed restraints; the optimal structures have minimal scores.

## 2a) Centromere localization

Fluorescence *in situ* hybridization (FISH) demonstrates that fission yeast interphase centromeres colocalize with the spindle pole body (SPB) diametrically opposite the nucleolus in ≥45% of populations that have been observed microscopically (3). Therefore, centromeres were modelled as being colocalized with the SPB, which was represented as a spherical region with a 200 nm radius (2–4) centered at $\boldsymbol{R}_{\boldsymbol{c}}$(*-1130,0,0*) or $\boldsymbol{R}_{\boldsymbol{c}}$(*-1510,0,0*) for the G1 and G2 models, respectively. The restraint is represented as a harmonic potential that acts only on granules representing centromeres$(i\in centromeres)$:

$U_{centromeres}=\sum_{i\in centromeres} \left\{ \begin{aligned} k_{c}{(\left| \boldsymbol{r}_{\boldsymbol{i}} \right.-\left. \boldsymbol{R}_{\boldsymbol{c}} \right|\boldsymbol{-}d\boldsymbol{)}}^{2}\boldsymbol{,}if \left| \boldsymbol{r}_{\boldsymbol{i}} \right.-\left. \boldsymbol{R}_{\boldsymbol{c}} \right|\boldsymbol{>}d \\ 0, otherwise \end{aligned} \right.$;

Where $\boldsymbol{r}_{\boldsymbol{i}}$is the vector of granule coordinates, $k_{c}$ is a harmonic potential strength constant,$d$ is the radius of the centromere spherical region and ***R****_c_* is the centre of the spherical region representing the centromeres as defined above. The value of $k_{c}$ is discussed below in the *Selection of harmonic potential strength constants* section.

## 2b) Telomere localization

Interphase telomeres cluster at the nuclear periphery (2). The telomere granules were restricted to the volume within *l* = 100 nm of the nuclear periphery (Figure 1):

$$U_{telomeres}=\sum_{i\in telomeres} \left\{ \begin{aligned} k_{t}{(R_{nucleus}-\left| \left. \boldsymbol{r}_{\boldsymbol{i}} \right| \right.\boldsymbol{-}l\boldsymbol{)}}^{2}\boldsymbol{,}if R_{nucleus}-\left| \left. \boldsymbol{r}_{\boldsymbol{i}} \right| \right.\boldsymbol{>}l \\ 0, otherwise \end{aligned} \right.;$$

where $\boldsymbol{r}_{\boldsymbol{i}}$is the vector of granule coordinates, $k_{t}$ is a harmonic potential strength constant,$l$ is the maximum distance from the nuclear membrane defining the restricted spherical layer and $R_{nucleus}$is the radius of nucleus. The value of $k_{t}$ is discussed below in the *Selection of harmonic potential strength constants* section.

## 2c) Nucleolus localization

The nucleolus, the site of ribosomal DNA (rDNA) transcription, forms a compartment in the nucleus that is positioned opposite the SPB (3). The nucleolus contains the two rDNA regions located in the subtelomeric regions on both arms of chromosome III(3). The previously-determined ratio of the nucleolus to nuclear volume${(V}_{nucleolus}=0.24 V_{nucleus})$(1) was used to calculate the nucleolus volume for the G1 and G2 models (Table 3).

**Table 3.** Nucleolar volumes for G1 and G2 phase models calculated using a nucleolus:nuclear volume ratio of 0.24 (1).

| **Cell cycle stage** | **Mutant name** | **Nuclear volume (μm^3^)** | **Nucleolus volume (μm^3^)** |
| --- | --- | --- | --- |
| G1 | cdc10-129 | 9.95 | 2.38 |
| G2 | cdc25-22 | 20.94 | 5.02 |

The organization of the rDNA repeats on chromosome III within the nucleolus is undetermined in the GCC data. Thus, we did not model the structure of the nucleolus in the polymer models.

The nucleolus compartment was modelled as an overlapping sphere of the same radius as the nucleus that was shifted to overlap the nuclear volume to create a nucleolus of the correct volume (Figure 1, Table 3).This gave the best approximation of the shape of the nucleolus as assessed by imaging (3).

All granules *except* those representing the rDNA $(i\neq rDNA)$ were excluded from the nucleolus using a repulsive harmonic potential:

$$U_{repulsive\_nucleolus}=\sum_{i\neq rDNA} \left\{ \begin{aligned} k_{rn}\left( \left| {\boldsymbol{r}_{\boldsymbol{i}}-\boldsymbol{R}}_{cn} \right|\boldsymbol{-}R_{nucleolus} \right)^{2}\boldsymbol{,}if \left| {\boldsymbol{r}_{\boldsymbol{i}}-\boldsymbol{R}}_{cn} \right|\boldsymbol{<}R_{nucleus} \\ 0, otherwise \end{aligned} \right.;$$

where$\boldsymbol{r}_{\boldsymbol{i}}$ is the vector of granule coordinates, $k_{rn}$ is a harmonic potential strength constant $\boldsymbol{R}_{\boldsymbol{cn}}$ is the coordinate vector for the centre of the shifted sphere and $R_{nucleus}$ and $R_{nucleolus}$are the radii of the nucleus and nucleolus, respectively. The value of $k_{rn}$ is discussed below in the *Selection of harmonic potential strength constants* section.

In addition, the terminal granules of chromosome III, which represent the rDNA array, were attracted to the nucleolus using an attractive harmonic potential:

$$U_{attractive\_nucleolus}=\sum_{i\in rDNA} k_{an}\left( \left| {\boldsymbol{r}_{\boldsymbol{i}}-\boldsymbol{R}}_{cn} \right|\boldsymbol{-}R_{nucleus} \right)^{2};$$

where$\boldsymbol{r}_{\boldsymbol{i}}$ is the vector of granule coordinates,$k_{an}$ is a harmonic potential strength constant, $\boldsymbol{R}_{\boldsymbol{cn}}$ is the coordinate vector of the center of the shifted sphere and $R_{nucleus}$ is the radius of the nucleus. For the G1 phase model$,$***R****_cn_* is (*1510,0,0*) and for the G2 model ***R****_cn_* is (*1941,0,0*). The terminal granules of chromosome III are the only granules that represent the rDNA in our models, as the genome sequence used (NC_003421) contained only three rDNA repeats on the left arm and two on the right arm of chromosome III.

## 2d) Chromatin persistence length

To reproduce the bending elasticity of chromosome fibres, we imposed an angular restraint between each set of three consecutive granules(14):

$$U_{bending}=\sum_{i\in N} T \frac{l_{p}}{s}(1-\cos\theta_{i});$$

where the sum runs over all possible sets of three consecutive granules, $T$is a temperature,$l_{p}$is the desired persistent length of 150nm (5), *θ* is the angle defined by the three granules and $s$is the size of granules in nm. $\cos\theta_{i}$was calculated as:

$$\cos\theta=\frac{(\boldsymbol{r}_{\boldsymbol{i+1}}-\boldsymbol{r}_{\boldsymbol{i}}\boldsymbol{)}(\boldsymbol{r}_{\boldsymbol{i+2}}-\boldsymbol{r}_{\boldsymbol{i+1}}\boldsymbol{)}}{\left| \boldsymbol{r}_{\boldsymbol{i+1}}-\boldsymbol{r}_{\boldsymbol{i}} \right|\left| \boldsymbol{r}_{\boldsymbol{i+2}}-\boldsymbol{r}_{\boldsymbol{i+1}} \right|};$$

where $\boldsymbol{r}_{\boldsymbol{i}}\boldsymbol{,}\boldsymbol{r}_{\boldsymbol{i+1}}\boldsymbol{,}\boldsymbol{r}_{\boldsymbol{i+2}}$are the vectors of coordinates for the three consecutive granules.

## 2e) GCC-derived chromosome interactions

The chromosome interaction data was obtained from the GCC data for cells synchronized in G1 and G2 phases of the cell cycle (this study). The GCC experimental data represents interactions between 9827 restriction fragments. To filter random ligation events, only significant non-adjacent interactions with frequencies ≥ 4 were used.

In order to be applied to the model as restraints, the GCC-derived data listing captured interactions between restriction fragments had to be converted to granule-granule interactions. Each AseI restriction fragment within the *S. pombe* genome was assigned to the granule that overlaps that region of the linear DNA sequence. In instances where the restriction fragment overlaps two or more granules, the granule containing the centre of the fragment was assigned as the interacting granule. In instances where multiple restriction fragments overlap a single granule, all interactions were assigned to that granule. As a result of this mapping procedure, it is possible that the granule-granule interaction list for any one granule may include repeats of interactions. In these cases, the strongest of the interaction repeats was retained.

GCC is a probabilistic method (15) that captures interactions within and between chromosomes in a population of cells at one moment in time. As such, the exact identities and numbers of interactions that occur within a single cell are unknown. Moreover, it remains possible that some captured interactions may not be simultaneously present in all cells and certain combinations could even be mutually exclusive, even in synchronized cells. To account for this, we incorporated subsets of the captured interactions into our models in a probabilistic manner. Specifically, to generate each structure, we used only 10% of the captured interactions randomly selected from the set of uniquely positioned GCC interactions. These interactions all occurred at frequencies above the experimental noise and were chosen with a probability proportional to their detection frequency. Thus, high frequency interactions have a higher probability of being included in individual structures. We calculated 500 structures based on different interaction subsets. Irrespective of the detection frequency, each uniquely positioned interaction was included in at least one of the 500 generated structures.

The GCC-derived chromosome interactions were incorporated into the model as a sum of attractive harmonic potentials:

$$U_{interactions}=\sum_{i\in interactions} k_{i}{(d_{i}-s)}^{2};$$

where$k_{i}$is a harmonic potential strength constant, $d_{i}$is the distance between interacting granules and$s$is the size of the granules in nm.

# Scoring function

The degree to which each structure fits the imposed restraints was defined by a scoring function or energy, *E*, with a smaller value of *E* indicating a better fit. The scoring function was defined as the sum of all of the two types of flexible restraint energies defined above:

$E=U_{tethering}+U_{bending}+U_{interactions}$;

${U_{tethering}=U}_{centromeres}+U_{telomeres}+U_{repulsive\_nucleolus}+U_{attractive\_nucleolus}$.

# Monte-Carlo approach for structure optimization

Scoring function (energy) minimization was performed using a Metropolis-Monte Carlo (MC) algorithm. The MC method is preceded by a sequence of random displacements of a part of the chromosome chain. Following each random displacement, the trial conformation is accepted or rejected according to the following criteria:

- If the energy (*E*) of the new conformation is lower than the energy of the previous conformation, $E_{new}<E_{old}$, the new conformation is accepted.
- If $E_{new}>E_{old},$the conformation is accepted according to the probability$p=exp(\frac{E_{old}-E_{new}}{T}).$ This prevents structures becoming stuck at a local rather than global minimum of *E* and determines the level of energy fluctuations in the system. If the new conformation is rejected, the system returns to the last accepted conformation.

# Selection of harmonic potential strength constants

Since the restraints are harmonic potentials ($U=k{\Delta x}^{2})$, the potential strength constant$k$ plays a crucial role with regard to the system energy variations. For a harmonic potential, the variation of system energy causes a variation of the distance from the centre of the potential $\Delta x=\sqrt{T/k}.$*T* was set to$37℃$, the temperature at which the experiments were carried out. For a desired distance variation range, the harmonic potential strength constant can therefore be calculated as $k=T/{(\Delta x)}^{2}$. For the strength constant calculation we selected the following distance variations:

- The restraint of granules representing centromeres, telomeres, and the start of the rDNA repeats to lie within particular regions of the nucleus was defined with $\Delta x=10 nm$resolution to ensure that these known biological constraints would be satisfied even after the introduction of GCC-derived interactions.
- The capture of interactions by GCC does not provide any information about what is mediating the interactions or the precise distances between the interacting fragments. It is possible that RNA polymerase II (50 nm diameter (16)) is a component of some interactions. Therefore, we assumed that the interaction distance may vary within the range 0-100 nm. Thus, $\Delta x$ was set to 100 nm (17) for the$U_{interactions}$potential energy function.

# Chain displacement algorithm

In each MC step one chromosome chain is randomly selected. A section of the selected chromosome is then chosen at random and subjected to displacement. The displacement is a random rotation about a randomly placed axis which is calculated using the Vologodskii and Rybenkov algorithm (14), which maintains the distances between the neighbouring granules.

Following the Vologodskii and Rybenkov algorithm (14) there are two types of chain segment displacements: *Type I* and *Type II*.

**A**

**B**


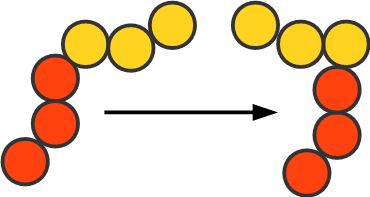

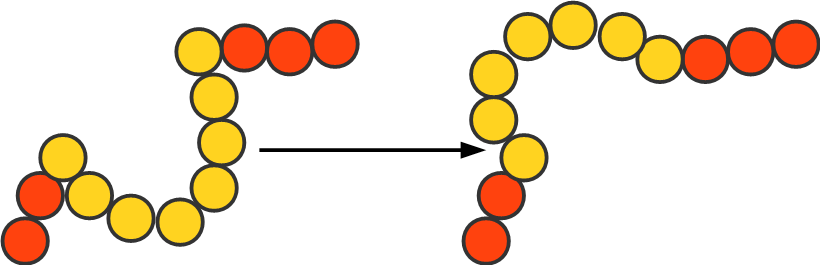


**Rotation axis**

**Direction of rotation**

**Figure 2** Cartoon diagram illustrating the difference between *Type I* and *Type II* displacements. A) *Type I* displacement: the section of chromosomes between two randomly selected granules rotates about an axis passing through the centers of the chosen granules. B) *Type II* displacement: one granule is selected and a randomly-oriented axis passing through this granule is generated. The shorter (terminal) part of the polymer chain is rotated about this axis.

## Type I displacement.

Two granules are chosen randomly on the same polymer chain and the axis of rotation is positioned to pass through the centre of these two granules. All the granules between the two chosen granules are rotated around this axis by a randomly determined angle (Figure 2).Thus, *Type I* displacement causes random deformation of the chain. However, *Type I* displacement does not move the terminal granules of the polymer chains.

## Type II displacement.

Type II displacement provides a mechanism for the movement of terminal granules on polymer chains. For a *Type II* deformation, a single granule is selected and the shorter (terminal) section of the polymer chain, starting from the selected granule, is randomly rotated about a randomly-oriented axis that is positioned to pass through the selected granule (Figure 2).

# Model generation procedure

## Step 1: Structure initialization

Initial chromosome positions were generated as follows. The coordinates of the first granule of each chromosome are randomly assigned to lie within the sphere representing the nucleus. The positions of the consecutive granules are calculated such that the distance between the centres of the granules equals the size of a granule, but the directions of the vectors $\boldsymbol{r}_{\boldsymbol{i+1}}-\boldsymbol{r}_{\boldsymbol{i}}$are uniformly distributed across the sphere. At this stage, the chain is a “phantom” and granules may overlap. At the end of the initial positioning of the chromosomes, some granules may lie outside the nuclear confinement.

## Step 2: Compress chromosomes within the nuclear confinement

Any granules lying outside the nucleus after initialization are moved to lie inside the nucleus by compressing the “phantom” chains. The chain displacement algorithm is applied, with the number of granules outside the confinement volume counted at each step. The structure is accepted if the number of granules outside confinement decreases or is unchanged, otherwise the structure is rejected. The compression procedure stops when all granules are enclosed in the nuclear confinement.

## Step 3: Turn on restraints

In this step the flexible restraints (Table 1) are introduced into the displacement of the “phantom” chains. Granule volume overlaps are still not included, because the calculation of new conformations that fit the restraints are the most time consuming part of the algorithm. Instead, the “phantom” chain conformations are determined and then the granule excluded volume is switched on. This approach improves the overall polymer mixing and saves calculation time. There are 5 × 10^6^ steps in this phase of chain displacement.

## Step 4: Turn on the granule excluded volume

At the beginning of this step, the “phantom” polymer chains that represent the chromosomes lie inside the nuclear confinement and fit the flexible restraints. However, the individual granules do not have any volume and can therefore overlap. To generate physically realistic, non-overlapping conformations, the excluded volume interactions are implemented. Because the immediate introduction of excluded volume could result in extremely high-energy conformations from which no acceptable displacements are possible, the excluded volume was turned on gradually.

Initially all granules were assigned spherical cores of 1 nm diameter. In addition to the scoring function values for “old” and “new” structures, the number of core overlaps was counted after each displacement move. If the number of overlapping cores reduced in the “new” structure then the “new” structure was accepted without taking into account the scoring function change. The “new” structure was rejected if the number of core overlaps increased. Finally, if the number of overlaps remained the same, then the models were accepted or rejected according to the usual MC acceptance rule based on the scoring function alone.

When the number of overlapping cores dropped to zero, the radius of each core was increased by 1 nm and the procedure repeated, until the granule radius reached 30 nm or 90 nm for the G1 and G2 models, respectively. Thus the final structures that were obtained maintained the architectural properties of the optimized conformation while incorporating granule excluded volume.

## Step 5: Final structure optimisation

The final stage of the modelling calculation is a second round of optimization of the scoring function. The previous step introduced excluded volume, while potentially increasing the model energy. Therefore, an additional 10^6^ simulation steps are performed to minimize the model energy.

# Model types

Three types of models, with different subsets of restraints, were developed. Note that all models incorporated excluded volume and the polymer chain persistence length.

- *Confined model.* A control in which chromosomes are randomly configured within the confinement of the nuclear environment, without any additional restraints.
- *Constrained model*. The chromosomes remain randomly configured within the confinement, but are additionally subjected to tethering restraints that constrain the positioning of the centromeres, telomeres and the rDNA (within the nucleolus).
- *Interactions model.* The chromosomes are confined and subjected to the tethering restraints and, additionally, the experimental GCC-derived inter- and intrachromosomal interactions.

# Representation of sister chromosome cohesion in the G2 model

Following replication in S phase, sister chromosomes are held together by a cohesin complex(6–8), until separation during the anaphase step (9, 18).This linkage of sister chromosomes by topological embrace imposes restraints on the independent movement of the chromosome copies. Therefore, sister chromosome binding restraints must be included in the G2 genome model in order to correctly model chromosome packing within the nucleus.

To investigate how far apart cohesin-linked chromosomes can move, models comprising pairs of chromosome II were generated. Each chromosome was modelled with 30 nm granules, each representing 3.5 Kb of DNA, giving 7166 granules in total. Sister chromosome linkages were incorporated at 228 cohesin complex binding sites along chromosome II (9). The midpoint coordinates of the 228 cohesin binding peaks were translated to the granule positions according to:

$granule=ceiling(\frac{Midpoint}{3.5Kbp})$.

The mean distance between cohesin binding sites along chromosome II is 13.1 Kb (median 16.5 Kb), thus 228 of the 1,289 granules of chromosome II were marked as cohesin binding sites. As a result, on average every fifth granule along chromosome II contained a cohesin binding site.

Sister chromosome II cohesin was incorporated into the model as a sum of attractive harmonic potentials that pulled each pair of cohesin binding granules together:

$$U_{cohesin}=\sum_{i\in cohesin binding sites} k_{cohesin}{(d_{i}-s)}^{2};$$

where$k_{cohesin}$is a harmonic potential strength constant, $d_{i}$is the distance between interacting granules, and $s$ is size of the granules in nm. Since the cohesin complex forms large rings (about 45-60nm in diameter), granules that contain cohesin binding sites contact each other, so the distance variation $\Delta x$ was set to 1nm.

We developed three types of models to quantify the separation of identical granules from different copies of chromosome II:

- ***Confined with cohesin.*** Two copies of chromosome II only are subjected to the excluded volume and persistent length restraint and cohesin binding restraints$U_{cohesion}$.
- ***Constrained with cohesin model.*** Two copies of all three chromosomes are confined to the nucleus and subjected to the excluded volume, persistence length and tethering restraints. Only the two copies of chromosome II are subjected to the cohesin restraints$U_{cohesion}$.
- ***Constrained without cohesin model*.** Two copies of all three chromosomes are confined to the nucleus and subjected to the excluded volume, persistence length and tethering restraints. There are no sister chromosome cohesin restraints imposed on chromosome II.

Twenty five structures were calculated using the procedure described above (Model generation procedure), for each of the three types of model (*Confined with cohesin, Constrained with cohesin, Constrained without cohesin*). The distances between identical granules from the different copies of chromosome II were calculated for each structure of each type of model. The cumulative distribution functions (CDF) for these inter-granule distances (Figure 3A) describe the probability that a particular distance is observed in each type of model.


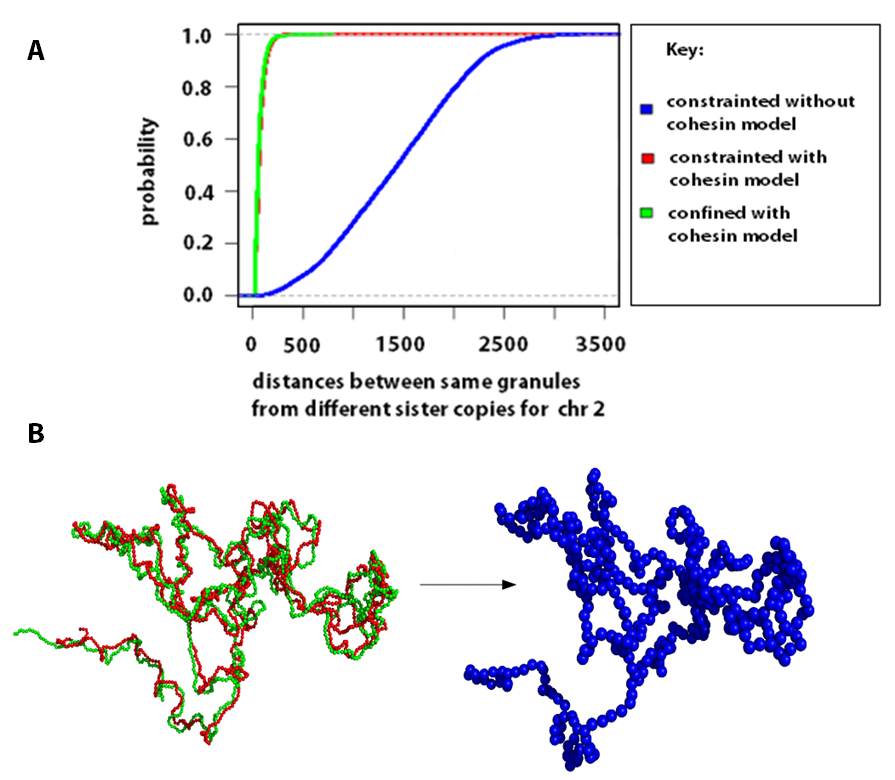


**Figure 3. Sister chromosomes in G2 phase are tightly linked together. (A)** The cumulative distribution functions (CDF) for distances between identical granules from the sister chromosomes showed that 50% of the granules in the *Constrained with cohesion* model were separated by less than 60nm. **(B)** Replacing two sister chromosomes, each 30nm in diameter and held together by cohesin, with one 90nm thick chromosome did not affect the polymer structure.

CDFs of the *Constrained with cohesin* and C*onstrained without cohesin* models indicate that the inclusion of the cohesin binding restraints significantly changes the distances between sister copies of chromosome II. For instance, 50% of the granules in the sister chromosomes were separated by less than 60nm in the *Constrained with cohesin model*. By contrast, 50% of the granules in the sister chromosomes were separated by 1400nm in the *Constrained without cohesin model*, indicating that the inclusion of cohesin restraints links the sister copies tightly together.

Based on this result, the two polymer chains representing the two copies of sister chromosomes in the *S. pombe* G2 cell cycle phase genome model were replaced by one thicker polymer. The size of the replacement chromosome granules was set to 90nm, calculated as the addition of the 50% separation value (60nm) to the diameter of the initial granule (30nm). Models of this 90nm thick polymer chain are very similar to those obtained for two linked 30 nm fibres (Figure 3B).

# Supplementary figures


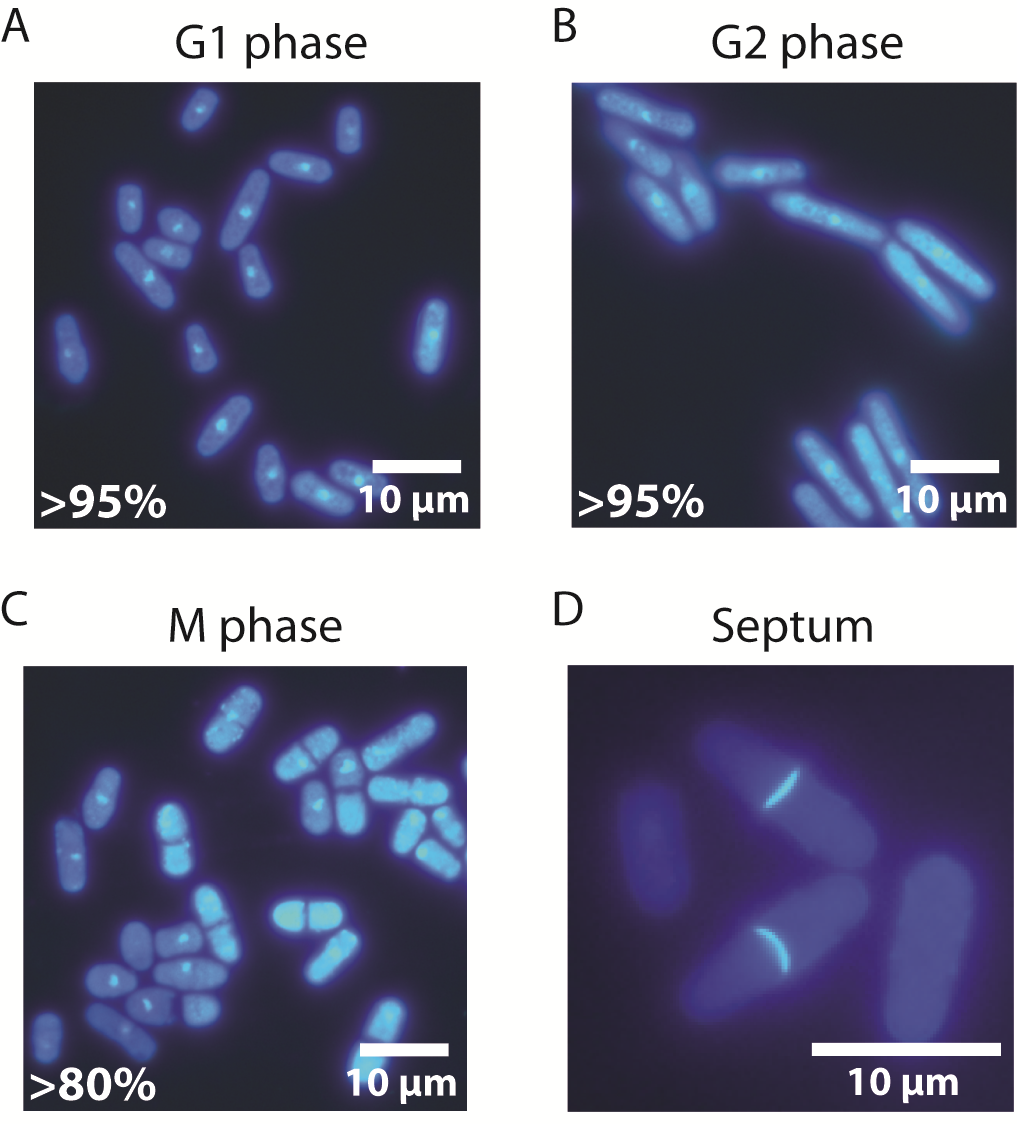


## Figure S1. Representative images of synchronized cells.

Photographs of *S. pombe* cells stained with calcofluor white were taken before and after synchronization using a fluorescence microscope. Representative images of synchronized cells are shown in: **(A)** G1 phase; **(B)** G2 phase; and **(C)** M phase. The numbers of cells with visible septa **(D)** were counted in at least 200 cells (total) from 10 fields of view. Cell cycle phase synchronization was calculated for the G1 and G2 phases by comparing the proportion of cells that had a visible septum in the pre-synchronized and synchronized cell populations.

## Figure S2. The captured chromosomal interactions were not fully saturated.

A collector’s curve was generated to determine whether the number of captured interactions between uniquely positioned loci (*i.e.* non-repetitive sequences) at each phase of the cell cycle reached a level of saturation with the depth of sequencing that was performed. The collector’s curves indicated that, despite the high correlation between biological replicates, the interaction network was not sampled to saturation. However, the high degree of correlation between the interaction networks detected for the biological replicates and the reduced correlations observed between the different cell cycle phases indicate that the most frequently occurring interactions were sampled. Moreover, the sampled interaction networks were significantly different from expected random distributions (Supplementary Table S6).


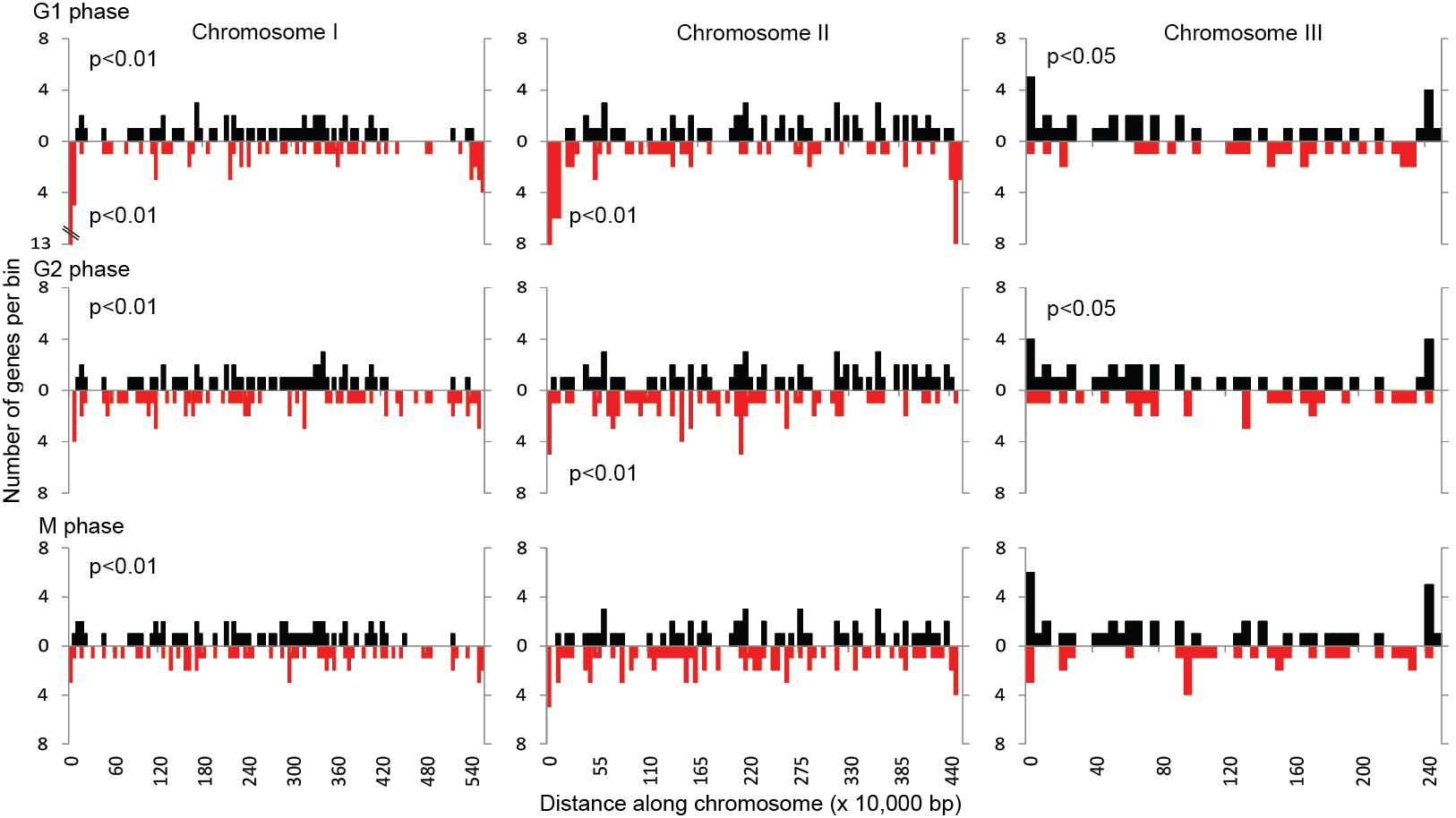


## Figure S3. Genes with high transcript levels were non-randomly distributed across one or more chromosomes at each stage of the cell cycle.

Genes with low transcript levels were predominantly non-randomly distributed along chromosomes only in the G1 phase. The chromosomal distribution of genes with high and low transcript levels was determined by calculating the central position (base pair) for each gene and using this to assign the genes along each chromosome into 50,000bp bins; **(Top)** G1 phase, **(Middle)** G2 phase, and **(Bottom)** M phase. One-sample Kolmogorov-Smirnov tests were performed using the non-binned chromosome distribution data to determine if highly and lowly transcribed genes were non-uniformly distributed across each chromosome (p-values are shown).


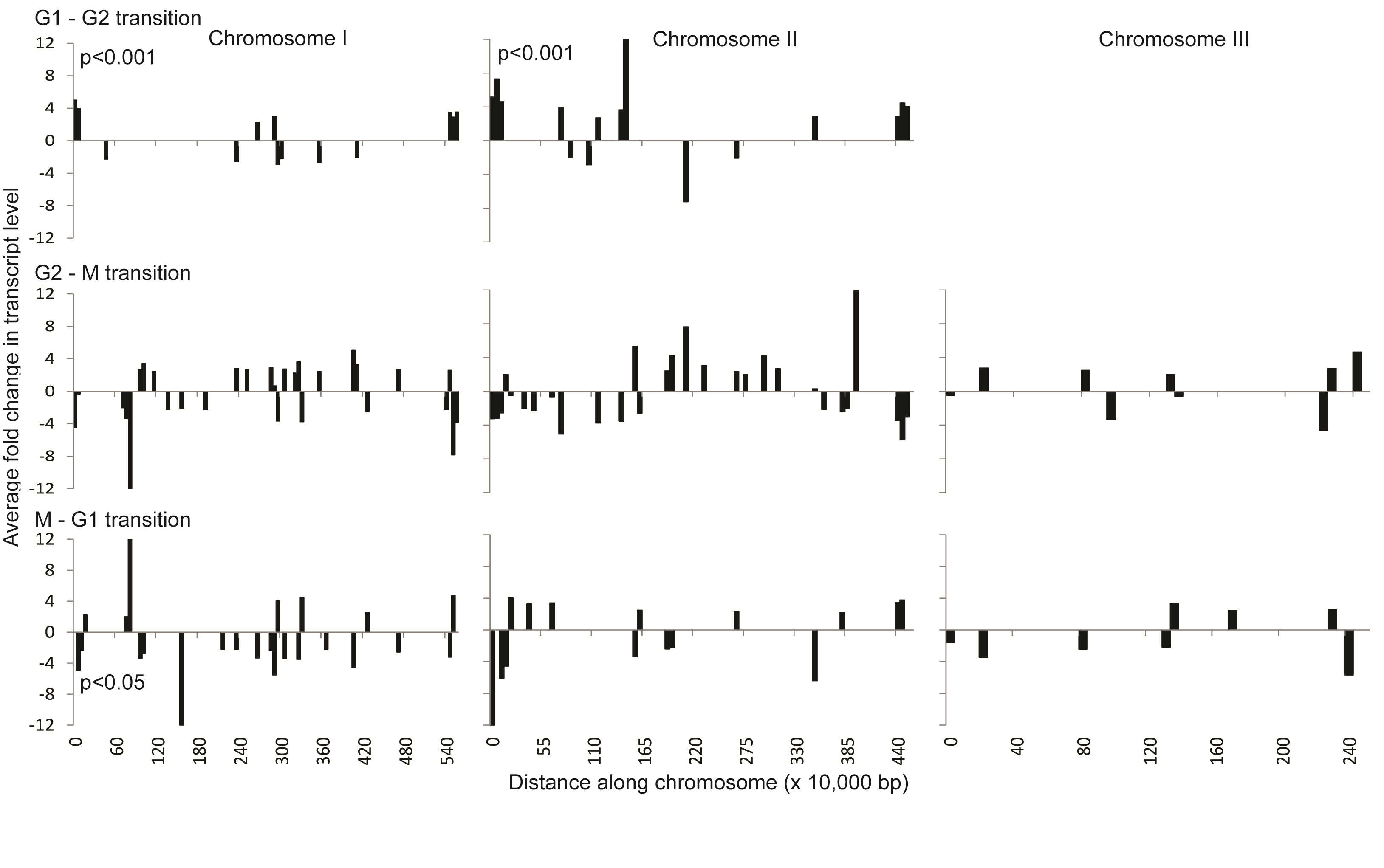


## Figure S4. Genes that were upregulated during the G1 – G2 transition and downregulated during the G2 – M transition had a highly significant non-random chromosomal distribution on chromosomes I and II.

The chromosomal positions of up- and downregulated genes (>2-fold change in transcript level) were used to calculate the average fold change in transcript level per bin (50 Kb) and depicted as histograms for the; **(Top)** G1→G2, **(Middle)** G2→M, and **(Bottom)** M→G1 cell cycle phase transitions. One-sample Kolmogorov-Smirnov tests were performed using the non-binned chromosome distribution data to determine if the differentially expressed genes were non-uniformly distributed across each chromosome.


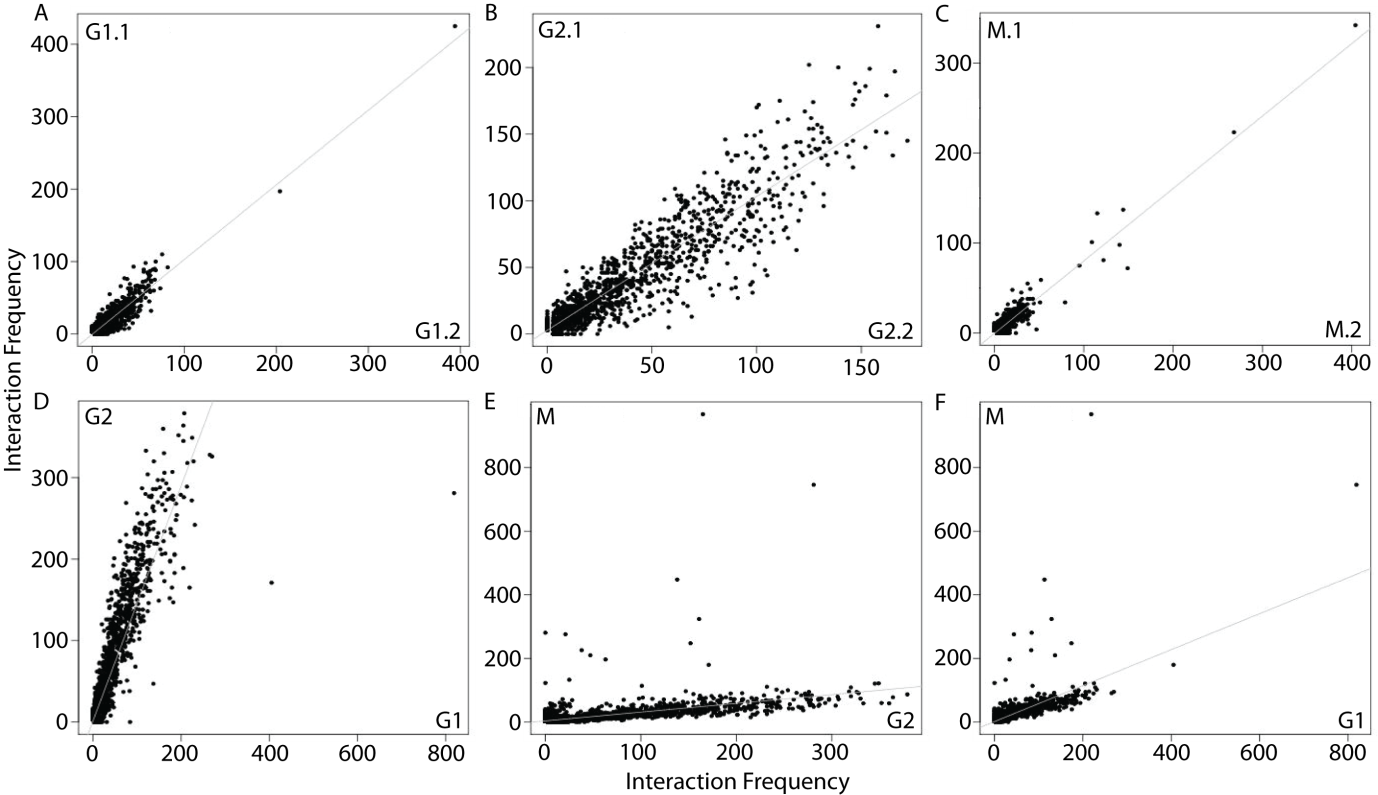
Figure S5. The chromosomal interactions that were captured for S. pombe were highly correlated between individual biological replicates and less correlated between the different cell cycle stages.

**(A-C)** The chromosomal interactions that were captured for the individual biological replicates were highly correlated at the AseI restriction fragment level. **(A)** G1 phase biological replicates (G1.1, replicate one verses G1.2, replicate two, R^2^=0.8643), **(B)** G2 phase biological replicates (G2.1, replicate one verses G2.2, replicate two, R^2^=0.8783), **(C)** M phase biological replicates (M.1, replicate one verses M.2, replicate two, R^2^=0.8692). **(D-F)** Interactions that were captured for the different stages of the cell cycle showed reduced correlation. **(D)** G1 verses G2 phase (R^2^=0.8058). The two outliers in the G1 phase involved LTR elements. **(E)** G2 verses M phase (R^2^=0.3239). **(F)** G1 verses M phase (R^2^=0.5335). Only uniquely positioned significant non-adjacent interactions were included in the correlation analysis.


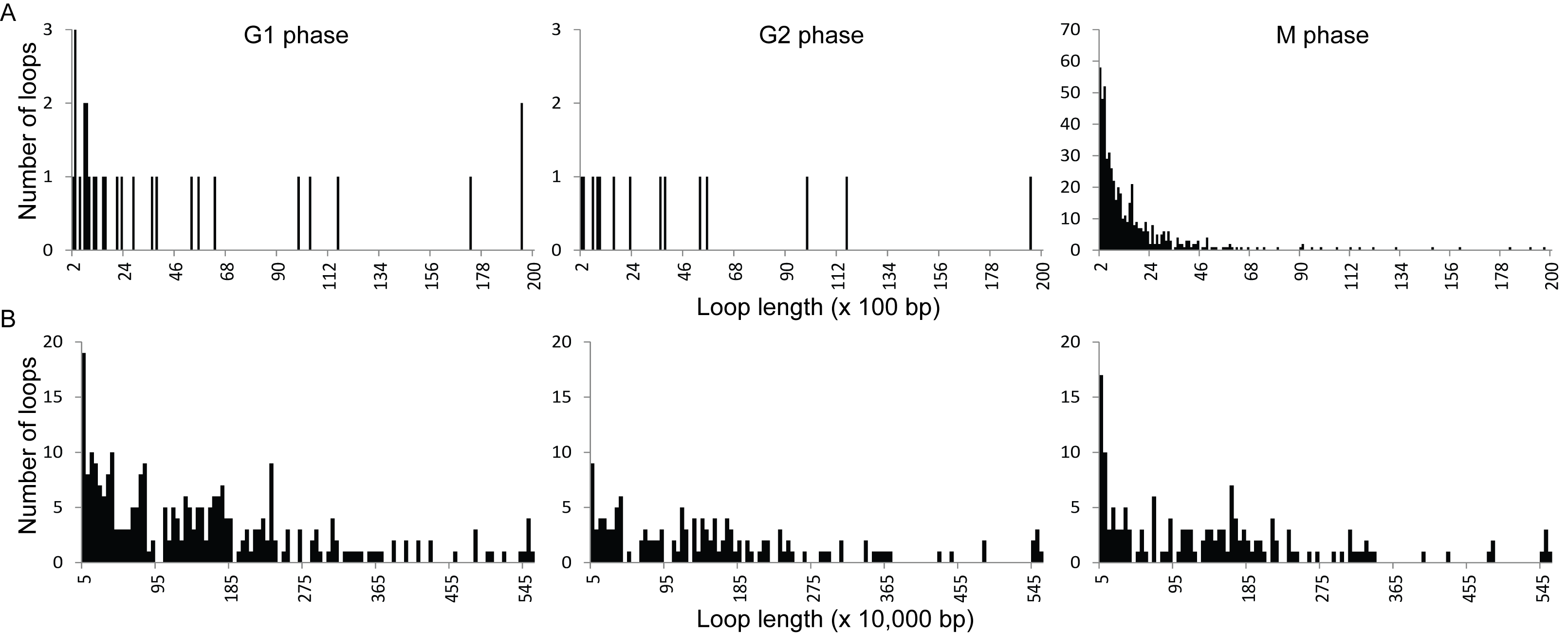


## Figure S6. There was a marked increase in the number of intrachromosome interactions with a loop length of ≤5 Kb that were captured in M phase.

The length of the loop (bp) between interacting genomic fragments was calculated and binned for all intrachromosomal interactions captured in each phase of the cell cycle. **(A)** Loop length frequencies plotted as a histogram with bin widths of 100 bp to highlight differences in the local structure. **(B)** Loop length frequencies plotted as a histogram with bin widths of 50 Kbp to highlight genome wide differences. The majority of loops were between fragments less than 200 bp apart in the linear sequence (G1 phase = 83.6%, G2 phase = 91.0%, and M phase = 68.1%). There was little difference in the proportion of distal (>50 Kb loop) intrachromosomal interactions between the different phases of the cell cycle (G1 phase = 85.4%, G2 phase = 92.0%, and M phase = 93.1%). For clarity, only loops with a length of ≤20 Kb are shown in **(A)** and interactions <200 bp are not shown in either **(A)** or **(B)**. Only uniquely positioned significant non-adjacent interactions were used for these analyses.

**
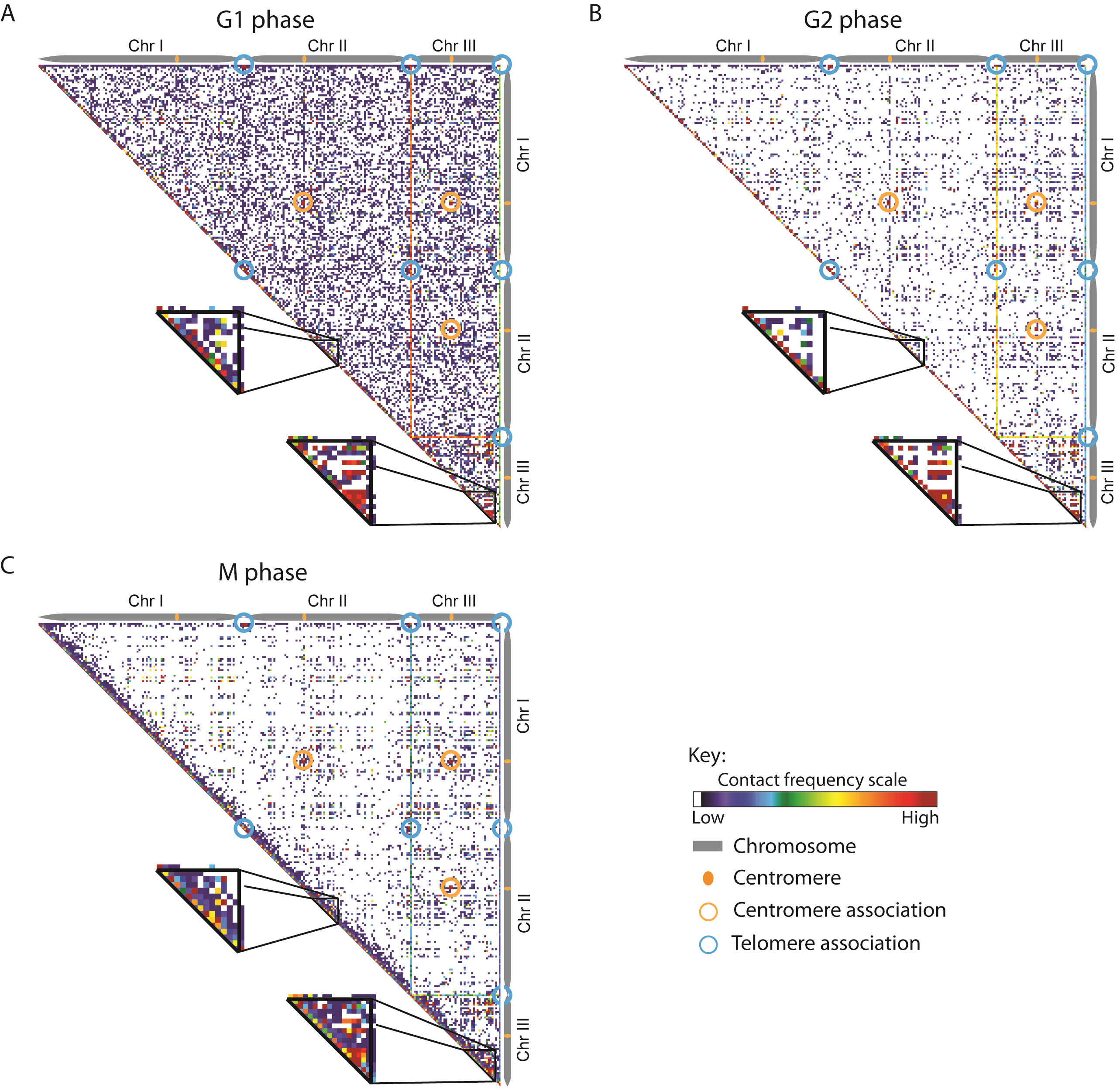
**Figure S7. Mapping of chromosome interactions throughout the fission yeast cell cycle.

All captured interactions were plotted on a chromosome contact map with 50 Kb resolution. TAD-like domains are present (highlighted on chromosomes II and III). The contact frequency is represented as the percentage of the total number of contacts that were captured (white = 0 red = ≥0.005%).


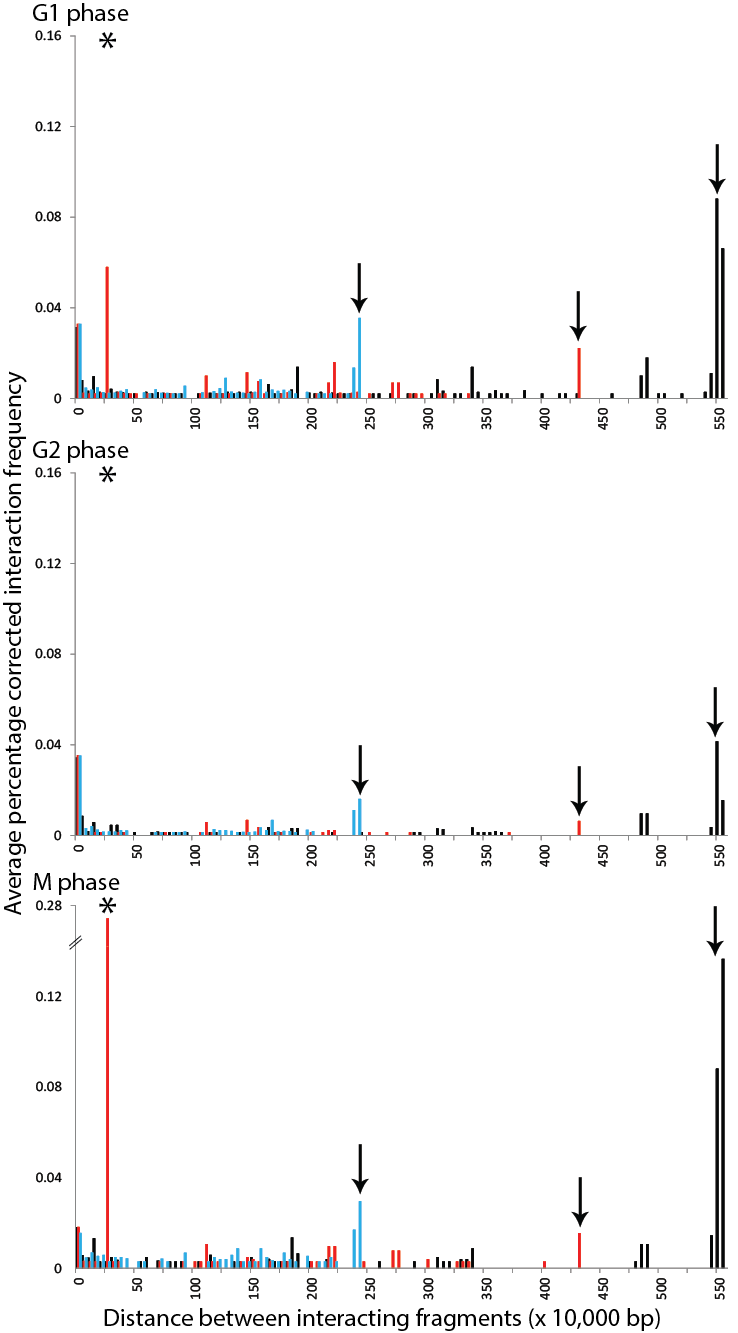


## Figure S8. The terminal sequences on the opposite arms of each chromosome colocalized to circularize each chromosome throughout the cell cycle.

Interaction frequency, expressed as a percentage of the total number of contacts, was plotted as a histogram for loop sizes grouped into 50 Kbp bins for each stage of the cell cycle: **(Top)** G1, **(Middle)** G2, and **(Bottom)** M phase. Results for the different chromosomes are coloured: chromosome I, black; II, red; III, blue. Arrows denote the loop lengths that indicate intrachromosomal telomere colocalization that results in the chromosomes assuming a circular conformation. An interaction between two fragments on chromosome II (Supplementary Table S8 and S9) fluctuated in frequency throughout the cell cycle (annotated by asterisks). Only uniquely positioned significant non-adjacent interactions were used for these analyses.


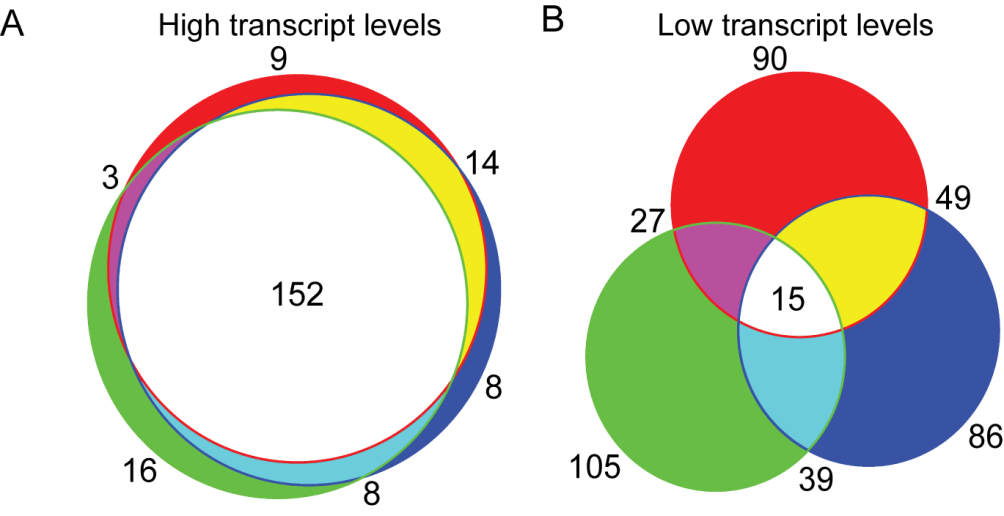


## Figure S9. Genes with high transcript levels in each cell cycle phase are highly conserved thoughout the cell cycle while genes with low transcript levels are predomenantly cell cycle phase specific.

**(A)** The majority of genes with high transcript levels were conserved at each cell cycle phase. **(B)** Genes with low transcript levels were largely specific to each cell cycle phase. The overlap between genes with the highest and lowest transcript levels at each phase of the cell cycle are displayed. Numbers represent the gene count in each subset. Here the RGB additive colour model (G1, red; G2, blue; and M, green) was used to highlight the intersections between the different gene sets.


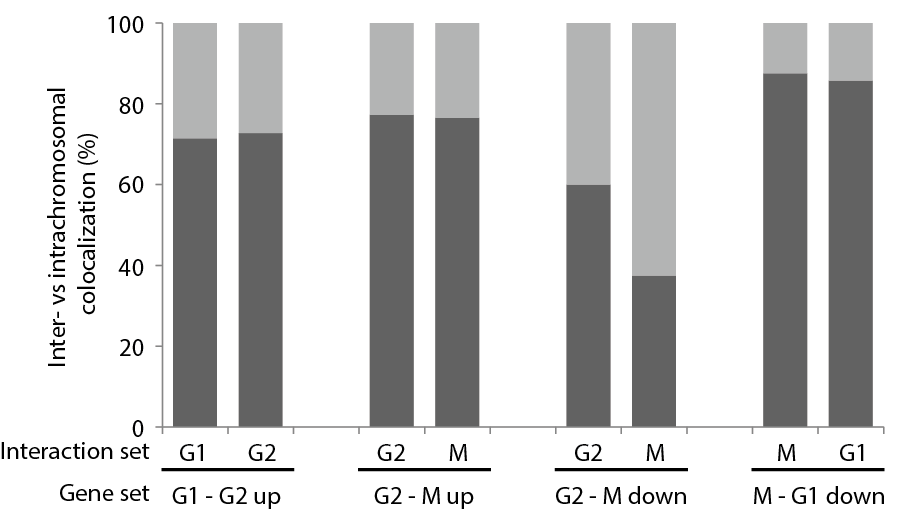


## Figure S10. The proportion of inter- versus intrachromosomal colocalization varied between genes with differential transcript levels.

Colocalization detected between differentially regulated genes (≥2-fold change in transcript level) was predominantly intrachromosomal, with the exception of G2→M downregulated genes. Furthermore, changes in intrachromosomal gene colocalization that correlated with differential gene expression were predominantly due to localized (>50 Kb) interactions (Supplementary Table S10). The percentage of inter- compared to intrachromosomal colocalization for genes with differential transcript levels was calculated and plotted for; G1→G2 and G2→M upregulated genes, and G2→M and M→G1 downregulated genes. The proportion of interchromosomal colocalization is depicted in light grey; the proportion of intrachromosomal colocalization is depicted in dark grey.


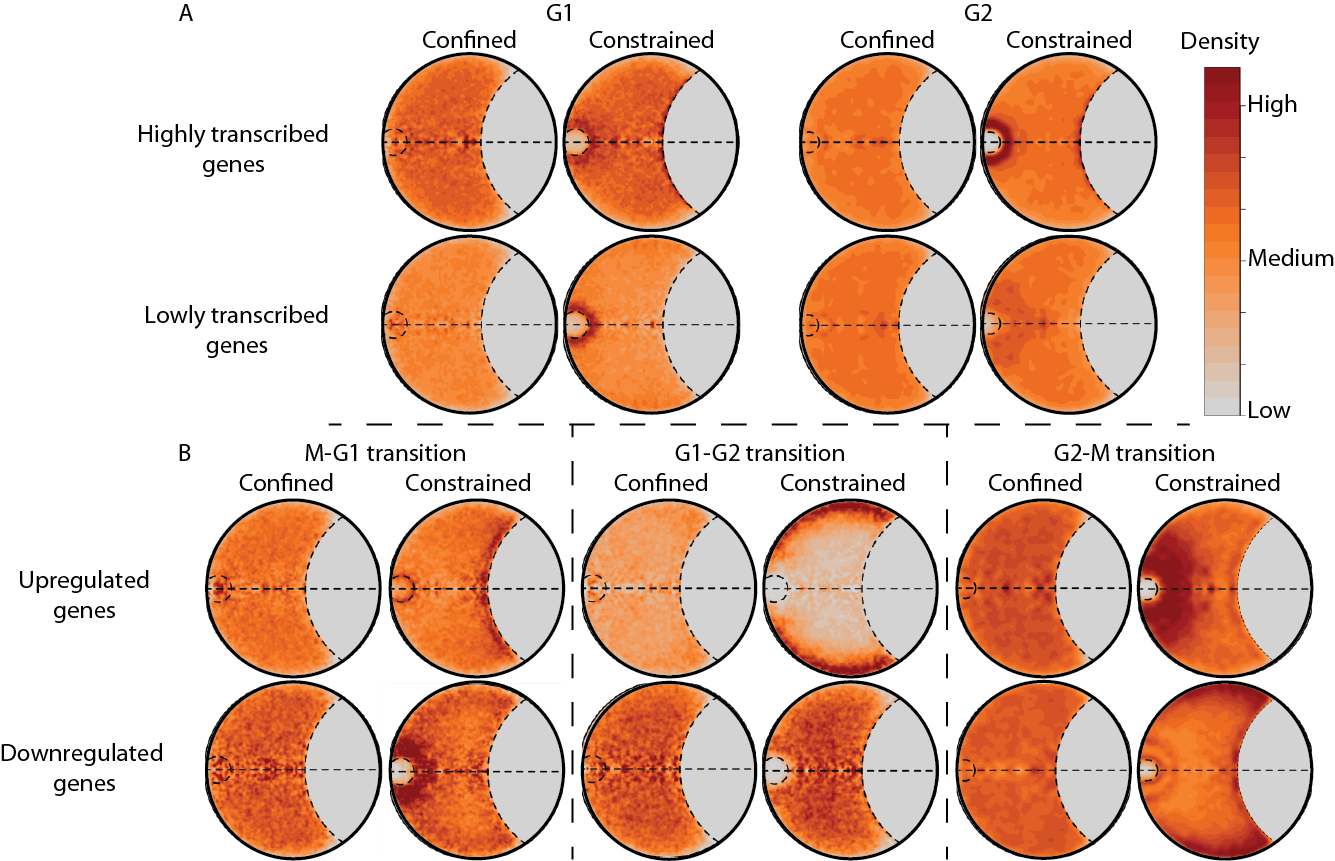


## Figure S11. Relative gene density maps reveal that the inclusion of tethering constraints (*i.e.* centromere association with SPB and telomere positioning at the nuclear periphery) is sufficient to provide a degree of order to sub-nuclear domains in G1 and G2 *S. pombe* nuclei.

**(A)** Relative density maps for genes with high and low transcript levels in the confined and constrained models for *S. pombe* nuclei during G1 and G2. **(B)** Relative density maps for genes that are upregulated during the M→G1, G1→G2, G2→M phase transitions in the confined and constrained models of the *S. pombe* G1 and G2 nucleus. The inclusion of interactions in the models made a significant difference to colocalization (see also Figure S13). Ensembles comprising 500 individual structures were generated using both the confined and the constrained models for each of the G1 and G2 cell cycle phase interaction sets. Genes with high, low, or differential transcript levels were mapped onto their respective granules, their relative density calculated for each structure and averaged across the ensemble. The positions of the genes that showed significant differences in transcript levels during the M→G1 and G1→G2 transition were mapped onto structures generated for the G1 phase. Genes that showed significant differences in transcript levels during the G2→M phase transition were mapped onto structures generated for the G2 phase of the cell cycle.


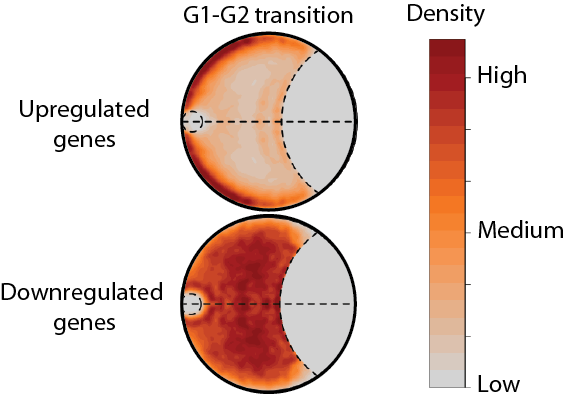


## Figure S12. Relative density maps for genes that are differentially regulated during the G1→G2 transition mapped onto the ensemble of structures generated using the interactions model for the G2 phase of the cell cycle.

An ensemble of 500 individual structures was generated using the interactions model and the G2 cell cycle phase interaction sets. Genes that showed significant differences in transcript levels during the G1→G2 phase transition were mapped onto their respective granules, the relative density of these granules in the nucleus averaged across the 500 models, and plotted as a gene density map.


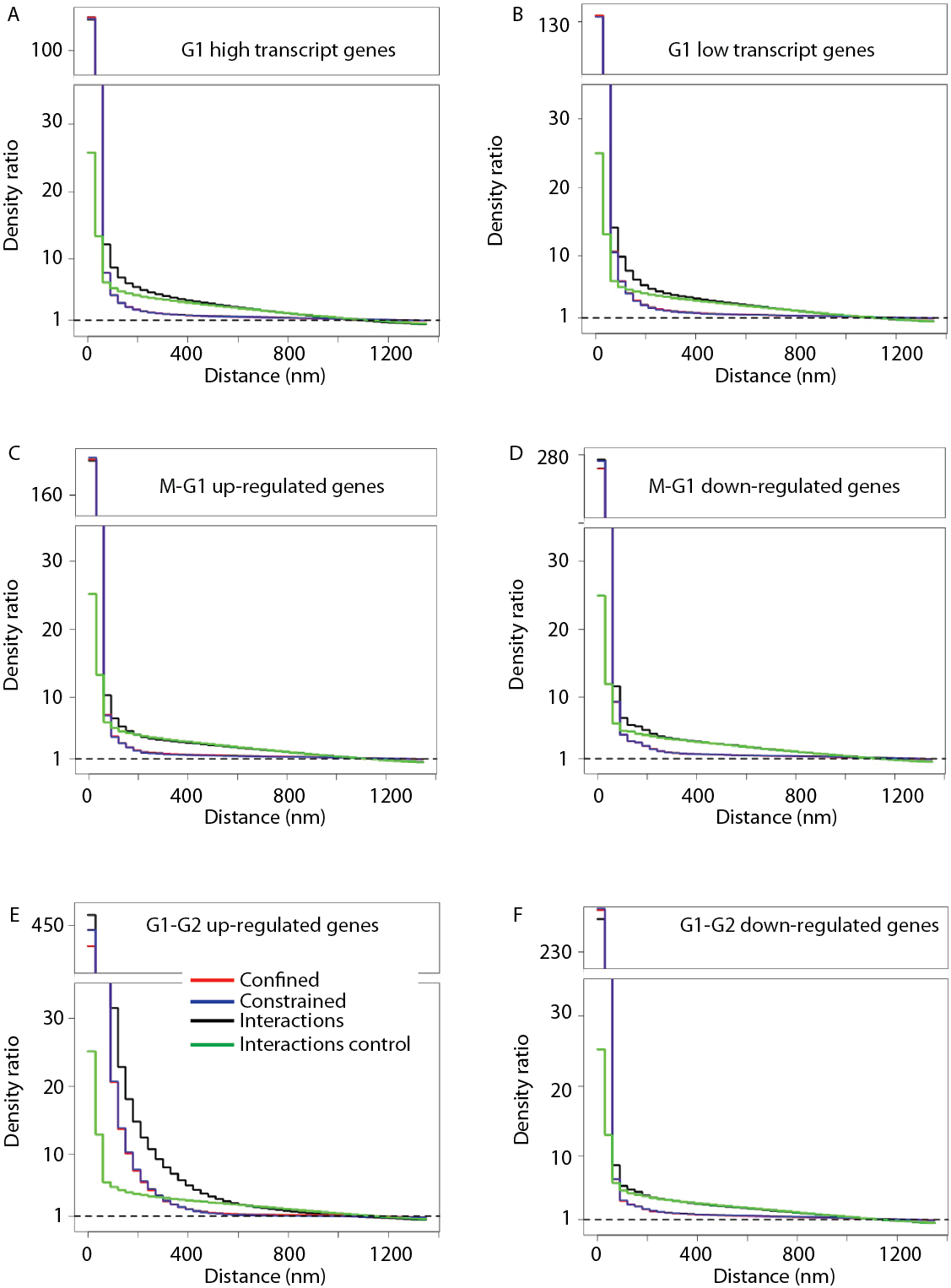


## Figure S13. Genes that are highly transcribed in G1 or upregulated during the M-G1 phase transition exhibit significant inter-gene clustering that is not explained by linear chromosome structure or general genome compaction.

Density distribution functions for genes **(A)** with high transcript levels in the G1 phase; **(B)** with low transcript levels in the G1 phase; **(C)** that are upregulated during the M→G1 phase shift; **(D)** that are downregulated during the M→G1 phase shift; **(E)** that are upregulated upon the G1→G2 phase shift; **(F)** that are downregulated upon the G1→G2 phase shift. DDFs were calculated for the ensemble of structures generated using the: (red) confined, (blue) constrained and (black) interactions models. DDFs were also determined for a set of randomly chosen loci (green line), rather than a specific set of genes, calculated from the interactions models, where the number of loci was equal to the number of genes under consideration. The broken black line depicts a density ratio of one. Note the DDFs for the confined (red) and constrained (blues) often overlap to a high degree.


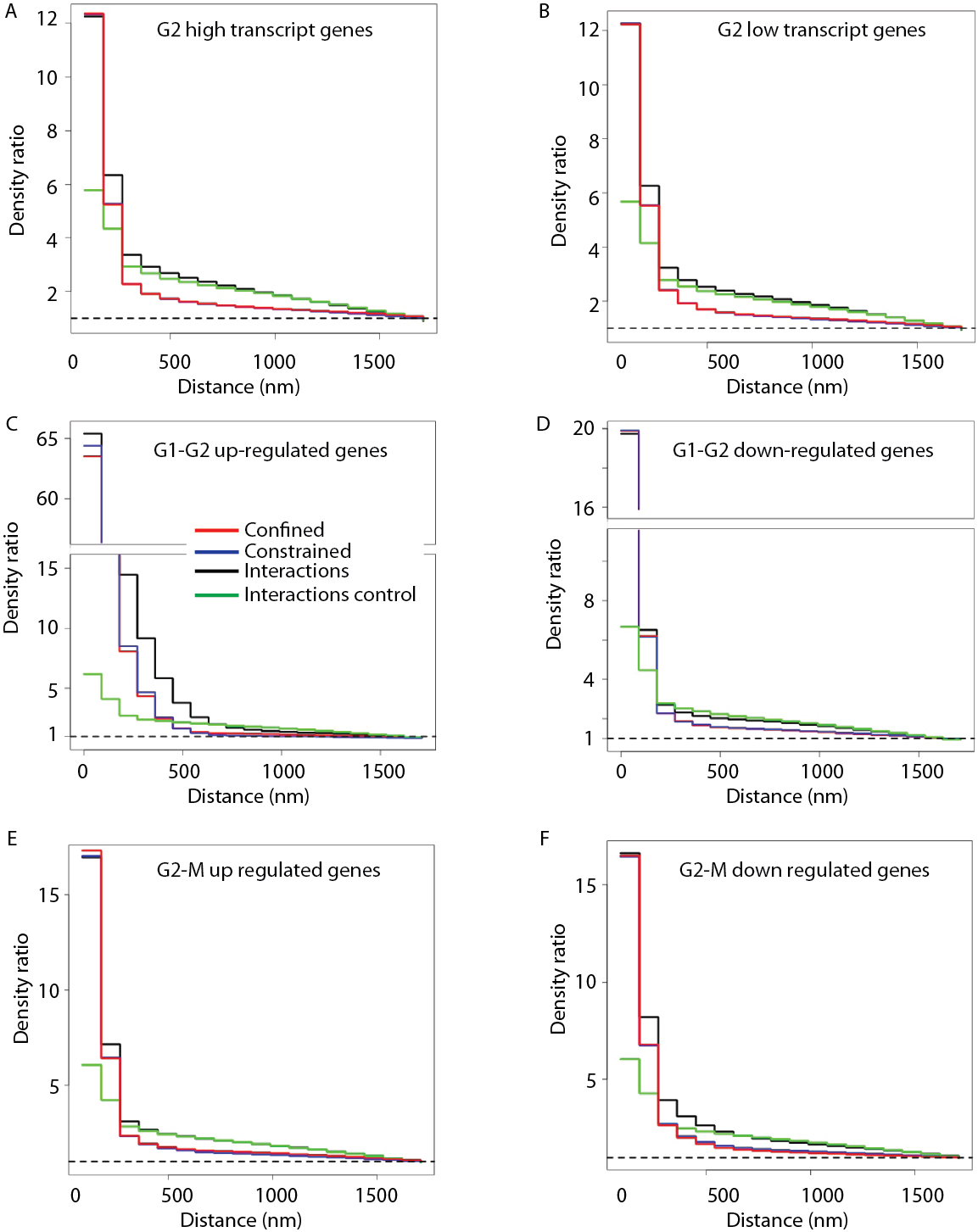


## Figure S14. Genes that are upregulated during the G1-G2 transition exhibit significant inter-gene clustering within the G2 phase models that is not explained by linear chromosome structure or general genome compaction.

Density distribution functions for genes **(A)** with high transcript levels in the G2 phase; **(B)** with low transcript levels in the G2 phase; **(C)** upregulated during the G1→G2 shift; **(D)** downregulated during the G1→G2 shift; **(E)** that are upregulated upon the G2→M phase shift; **(F)** that are downregulated upon the G2→M phase shift. DDFs were calculated for the ensemble of structures generated using the: (red) confined, (blue) constrained and (black) interactions models. DDFs were also determined for a set of randomly chosen loci (green line), rather than a specific set of genes, calculated from the interactions models, where the number of loci was equal to the number of genes under consideration. The broken black line depicts a density ratio of one.

# Supplementary tables

| Strain Name | Genotype | Reference |
| --- | --- | --- |
| MY291 | h- lue1 cdc10-129 | (19) |
| MY284 | h- lue1 cdc25-22 | (20) |
| MY286 | h- lue1 nuc2-663 | (21) |

## Table S1. *Schizosaccharomyces pombe* strains used in this study.

All strains were obtained from the National BioResource Project – Yeast (<http://yeast.lab.nig.ac.jp/nig/index_en.html>).

| G2 phase (cdc25-22) biological replicate #1 | | |
| --- | --- | --- |
|  | Before synchronization | After synchronization |
| Total cells counted | 225 | 204 |
| Number with a visible septum | 48 | 2 |
| Percentage | 21.33 | 0.98 |
| Synchronization efficiency | 100 – ((0.98 / 21.33) x 100) = **95.41%** | |

## Table S2. The synchronization efficiency for each of the G1 and G2 cell cycle phase biological replicates was calculated by comparing the proportion of cells with a septum before and after synchronization.

An example calculation of the cell culture synchronization efficiency for one of the G2 phase biological replicates is shown.

| Primer name | Sequence | Length of product (bp) |
| --- | --- | --- |
| E.coli191bp3’AseIF | TAGGCAGGATAAGGCGTTCA | 191 |
| E.coli191bp3’AseIR | GTGATTAATGCGGTCTGATGAGTCGTTTC |  |
| pRS426_185bp3’AseIF | TTGGTCTGACAGTTACCAATGC | 185 |
| pRS426_185bp3’AseIR | GTGATTAATGATAAATCTGGAGCCGGTGA |  |
| Lambda187bp3’AseIF | TTTACAGCGTGATGGAGCAG | 187 |
| Lambda187bp3’AseIR | GTGATTAATACCAATCCAGCCGGTCAG |  |

## Table S3. Ligation controls used in this study. Three short DNA sequences were amplified from the *E. coli* genome, pRS426 plasmid, and Lambda phage DNA.

An AseI site (red) was introduced into each amplicon within the reverse (AseIR) primer sequence. PCR amplicons were purified, digested with AseI and introduced into the GCC samples (at a 1:1 ratio with genome/cell number) before ligation to control for random inter-molecular ligation events.

| Cell cycle phases | G1 phase | G2 phase | M phase |
| --- | --- | --- | --- |
| Number of chromosome loci that contained genes with high transcript levels | 178 | 182 | 179 |
| Number of chromosome loci that contained genes with low transcript levels | 181 | 189 | 186 |

## Table S4. Table highlighting the number of chromosomal loci that had the highest (top 5%) and lowest (bottom 5%) transcript levels at each cell cycle phase and whether they were differentially regulated during cell cycle transitions.

Following analysis by Cufflinks, the genes that had the highest (top 5%) and lowest (bottom 5%, excluding genes that were not expressed) transcript levels were determined for each phase of the cell cycle.

| Cell cycle phase transitions | G1 → G2 phase | G2 → M phase | M → G1 phase |
| --- | --- | --- | --- |
| Total number of genes differentially expressed | 198 | 346 | 239 |
| Number of significantly upregulated genes  (percentage of total) | 102  (51.51%) | 138  (39.88%) | 150  (62.76%) |
| Number of significantly downregulated genes  (percentage of total) | 96  (48.49%) | 208  (60.12%) | 89  (37.24%) |
|  | | | |
| Genes with a fold change in transcript level ≥2 | 91 | 142 | 70 |
| Number of genes upregulated (cut-off ≥2)  (percentage of total) | 77  (84.62%) | 46  (32.39%) | 26  (37.14%) |
| Number of genes downregulated (cut-off ≥-2)  (percentage of total) | 14  (15.38%) | 96  (67.61%) | 44  (62.86%) |

## Table S5. The number of genes that were significantly differentially regulated during each *S. pombe* cell cycle transition.

RNA-seq data was analysed using Cufflinks (22, 23) to identify genes that were significantly up- and downregulated during each *S. pombe* cell cycle transition: G1 → G2 phase, G2 → M phase, and M → G1 phase. The total number of genes, and those that had a ≥2-fold change in transcript level, are displayed.

| **Non-reducing random sampling** | |
| --- | --- |
| All significant non-adjacent interactions | p = <0.001 |
| Significant interchromosomal interactions | p = <0.001 |
| Significant non-adjacent intrachromosomal interactions | p = <0.001 |
| **Reducing random sampling** | |
| All significant non-adjacent interactions | p = <0.001 |
| Significant interchromosomal interactions | p = <0.001 |
| Significant non-adjacent intrachromosomal interactions | p = <0.001 |

## Table S6. The sampled network is significantly different from that expected for randomly selected interactions.

Random networks were generated from a non-reducing and reducing pool of the total captured interactions. Chi-squared tests were performed to compare the average number of significant specific and shared interactions detected in the randomly selected sets with the actual experimentally determined interaction sets.

| **Interaction set** | **p value**  **CR** | **p value**  **R** |
| --- | --- | --- |
| G1 phase specific inter | <0.001 | <0.001 |
| G1 phase shared inter G1 G2 | 0.002 | 0.004 |
| G2 phase shared inter G1 G2 | 0.006 | 0.002 |
| G2 phase specific inter | <0.001 | <0.001 |
| G2 phase shared inter G2 M | <0.001 | <0.001 |
| M phase shared inter G2 M | 0.001 | 0.001 |
| M phase specific inter | <0.001 | <0.001 |
| M phase shared inter M G1 | <0.001 | <0.001 |
| G1 phase shared inter M G1 | <0.001 | <0.001 |
| G1 phase shared inter G1 G2 M | 0.028 | 0.027 |
| G2 phase shared inter G1 G2 M | 0.019 | 0.025 |
| M phase shared inter G1 G2 M | 0.021 | 0.022 |
| G1 phase specific intra | <0.001 | <0.001 |
| G1 phase shared intra G1 G2 | 0.001 | 0.005 |
| G2 phase shared intra G1 G2 | 0.005 | 0.010 |
| G2 phase specific intra | 0.008 | 0.014 |
| M phase specific intra | 0.001 | <0.001 |
| M phase shared intra M G1 | <0.001 | <0.001 |
| G1 phase shared intra M G1 | 0.001 | <0.001 |

## Table S7, p values for significant LTR colocalization sets for Figure 2.

Inter, interchromosomal; intra, intrachromosomal; CR, conserved random; R, random clustering (see methods).

| Chromosme (Fragment ID) | Coordinates (bp) | Fragment length (bp) |
| --- | --- | --- |
| Chr II (F1) | 2,163,364 – 2,164,371 | 1,007 |
| Chr II (F2) | 2,423,879 – 2,432,127 | 9,000 |

## Table S8. Chromosomal coordinates and lengths of the restriction fragments involved in a high frequency intrachromosomal interaction detected within chromosome II (Supplementary Figure S4).

Both fragments contain an LTR element.

|  | Fold change in transcript level | | |
| --- | --- | --- | --- |
| Fragment ID and overlapping genes | G1 → G2 phase (significant) | G2 → M phase (significant) | M → G1 phase (significant) |
| F2: Ubiquitin-protein ligase E3 (SPBC21D10.09c) | -0.3075 (no) | 0.9183 (yes) | -0.6108 (no) |

## Table S9. The absence of the interaction between two LTR elements in chromosome II during G2 phase was associated with the up regulation of the overlapping ubiquitin-protein ligase E3 gene.

The ubiquitin-protein ligase E3 gene was internal to and spanned ~70% of fragment F2 (Table S7).

| Gene set | % intrachromosomal  colocalization between elements >50 Kb apart | |
| --- | --- | --- |
| G1 → G2 upregulated | G1: 40 | G2: 62.5 |
| G2 → M upregulated | G2: 0 | M: 0 |
| G2 → M downregulated | G2: 41.7 | M: 14.3 |
| M → G1 downregulated | M: 0 | G1: 0 |
| G1 high transcript | 93.7 | |
| G1 low transcript | 57.1 | |
| G2 high transcript | 86.7 | |
| G2 low transcript | 76.9 | |
| M high transcript | 84.2 | |
| M low transcript | 81.8 | |

## Table S10. There was a reduction in intrachromosomal colocalization of differentially regulated genes that were >50 Kb apart.

This was particularly noticeable for genes that exhibited differential changes in transcript levels around the M phase.

| **Interaction set** | **Gene set** | **p value**  **CR** | **p value**  **R** |
| --- | --- | --- | --- |
| G1 phase shared inter G1 G2 | shared high transcript levels | 0.001 | <0.001 |
| G2 phase shared inter G1 G2 | shared high transcript levels | 0.001 | <0.001 |
| M phase shared inter M G1 | shared high transcript levels | 0.010 | 0.008 |
| G1 phase shared inter M G1 | shared high transcript levels | 0.001 | 0.001 |
| G1 phase shared inter G1 G2 M | shared high transcript levels | <0.001 | <0.001 |
| G2 phase shared inter G1 G2 M | shared high transcript levels | 0.003 | 0.002 |
| M phase shared inter G1 G2 M | shared high transcript levels | 0.008 | 0.006 |
| G1 phase specific intra | shared high transcript levels | 0.014 | 0.003 |
| G2 phase shared intra G1 G2 M | shared high transcript levels | 0.019 | 0.023 |
| G1 phase shared inter G1 G2 | G1 low transcript levels | 0.028 | 0.026 |
| M phase specific intra | M low transcript levels | 0.005 | 0.001 |
| G1 phase shared intra G1 G2 M | G1 low transcript levels | 0.033 | 0.034 |
| G2 phase shared intra G1 G2 M | G1 low transcript levels | 0.004 | 0.013 |
| M phase shared intra G1 G2 M | G1 low transcript levels | 0.016 | 0.006 |

## Table S11, p values for significant colocalization of genes with high and low transcript levels for Figure 4.

Inter, interchromosomal; intra, intrachromosomal; CR, conserved random; R, random clustering (see methods).

| **Interaction set** | **Gene set** | **p value**  **CR** | **p value**  **R** |
| --- | --- | --- | --- |
| G1 phase specific inter | G1 G2 up regulated | 0.011 | 0.028 |
| G1 phase shared inter G1 G2 | G1 G2 up regulated | 0.003 | 0.004 |
| G2 phase shared inter G1 G2 | G1 G2 up regulated | 0.002 | 0.002 |
| G1 phase shared intra G1 G2 | G1 G2 up regulated | 0.005 | 0.002 |
| G2 phase shared intra G1 G2 | G1 G2 up regulated | 0.006 | 0.009 |
| G2 phase specific intra | G1 G2 up regulated | 0.024 | 0.016 |
| M phase shared inter M G1 | M G1 down regulated | 0.008 | 0.009 |
| G1 phase shared inter M G1 | M G1 down regulated | 0.012 | 0.007 |
| G2 phase specific intra | G2 M down regulated | 0.031 | 0.031 |
| M phase shared intra G1 G2 M | G2 M down regulated | 0.004 | 0.001 |
| G1 phase shared intra G1 G2 M | M G1 down regulated | 0.049 | 0.046 |
|  |  |  |  |

## Table S12, p values for significant colocalization sets for Figure 5.

Inter, interchromosomal; intra, intrachromosomal; CR, conserved random; R, random clustering (see methods).

**References**

1. Neumann, F.R. and Nurse, P. (2007) Nuclear size control in fission yeast. *J. Cell Biol.*, **179**, 593−600.

2. Funabiki, H., Hagan, I., Uzawa, S. and Yanagida, M. (1993) Cell cycle-dependent specific positioning and clustering of centromeres and telomeres in fission yeast. *J. Cell Biol.*, **121**, 961–976.

3. Iwasaki, O., Tanaka, A., Tanizawa, H., Grewal, S.I.S. and Noma, K.I. (2010) Centromeric localization of dispersed Pol III genes in fission yeast. *Mol. Biol. Cell*, **21**, 254–265.

4. Kniola, B., O’Toole, E., McIntosh, J.R., Mellone, B., Allshire, R., Mengarelli, S., Hultenby, K. and Ekwall, K. (2001) The domain structure of centromeres is conserved from fission yeast to humans. *Mol. Biol. Cell*, **12**, 2767–75.

5. Bystricky, K., Heun, P., Gehlen, L., Langowski, J. and Gasser, S.M. (2004) Long-range compaction and flexibility of interphase chromatin in budding yeast analyzed by high-resolution imaging techniques. *Proc Natl Acad Sci U S A*, **101**, 16495–16500.

6. Haering, C.H., Farcas, A.-M., Arumugam, P., Metson, J. and Nasmyth, K. (2008) The cohesin ring concatenates sister DNA molecules. *Nature*, **454**, 297–301.

7. Haering, C.H., Löwe, J., Hochwagen, A. and Nasmyth, K. (2002) Molecular architecture of SMC proteins and the yeast cohesin complex. *Mol. Cell*, **9**, 773–88.

8. Hirano, T. (2006) At the heart of the chromosome: SMC proteins in action. *Nat. Rev. Mol. Cell Biol.*, **7**, 311–22.

9. Schmidt, C.K., Brookes, N., Uhlmann, F. and Christine K Schmidt, N.B. and F.U. (2009) Conserved features of cohesin binding along fission yeast chromosomes. *Genome Biol.*, **10**, R52.

10. Gehlen, L.R., Gruenert, G., Jones, M.B., Rodley, C.D., Langowski, J.. and O’Sullivan, J.M. (2012) Chromosome positioning and the clustering of functionally related loci in yeast is driven by chromosomal interactions. *Nucleus*, **3**, 1–15.

11. Wong, H., Marie-nelly, H., Herbert, S., Carrivain, P., Blanc, H., Koszul, R., Fabre, E. and Zimmer, C. (2012) A predictive computational model of the dynamic 3D interphase yeast nucleus. *Curr. Biol.*, **22**, 1881–90.

12. Tjong, H.A., Gong, K.A., Chen, L.A. and Alber, F.A. (2012) Physical tethering and volume exclusion determine higher-order genome organization in budding yeast. *Genome Res.*, **22**, 1295–1305.

13. Rapaport, D.C. (2007) The art of molecular dynamics simulation Cambridge University Press, New York.

14. Vologodskii, A. and Rybenkov, V. V (2009) Simulation of DNA catenanes. *Phys. Chem. Chem. Phys.*, **11**, 10543–52.

15. O’Sullivan, J., Hendy, M., Pichugina, T., Wake, G. and Langowski, J. (2013) The statistical-mechanics of chromosome conformation capture. *Nucl. (United States)*, **4**, 1–9.

16. Cramer, P., Bushnell, D. a and Kornberg, R.D. (2001) Structural basis of transcription: RNA polymerase II at 2.8 angstrom resolution. *Science*, **292**, 1863–76.

17. Rickman, C. and Bickmore, W. a (2013) Transcription. Flashing a light on the spatial organization of transcription. *Science*, **341**, 621–2.

18. Ocampo-Hafalla, M.T. and Uhlmann, F. (2011) Cohesin loading and sliding. *J. Cell Sci.*, **124**, 685–91.

19. Aves, S.J., Durkacz, B.W., Carr, A. and Nurse, P. (1985) Cloning, sequencing and transcriptional control of the Schizosaccharomyces pombe cdc10 “start” gene. *EMBO J.*, **4**, 457–463.

20. Nurse, P., Thuriaux, P. and Nasmyth, K. (1976) Genetic control of the cell division cycle in the fission yeast Schizosaccharomyces pombe. *Mol. Gen. Genet.*, **146**, 167–178.

21. Hirano, T., Hiraoka, Y. and Yanagida, M. (1988) A temperature-sensitive mutation of the Schizosaccharomyces pombe gene nuc2+ that encodes a nuclear scaffold-like protein blocks spindle elongation in mitotic anaphase. *J. Cell Biol.*, **106**, 1171–1183.

22. Trapnell, C., Hendrickson, D.G., Sauvageau, M., Goff, L., Rinn, J.L. and Pachter, L. (2012) Differential analysis of gene regulation at transcript resolution with RNA-seq. *Nat. Biotechnol.*, **31**, 46–53.

23. Trapnell, C., Roberts, A., Goff, L., Pertea, G., Kim, D., Kelley, D.R., Pimentel, H., Salzberg, S.L., Rinn, J.L. and Pachter, L. (2012) Differential gene and transcript expression analysis of RNA-seq experiments with TopHat and Cufflinks. *Nat. Protoc.*, **7**, 562–78.
