# Supplementary material for: Chromosome conformation maps in fission yeast reveal cell cycle dependent sub nuclear structure
Source: Nucleic Acids Res. 2014 Oct 23;42(20):12585–99. doi: 10.1093/nar/gku965 (PMC4227791; doi:10.1093/nar/gku965)
Supplement: SUPPLEMENTARY DATA [file supp_gku965_nar-01186-n-2014-File009.zip › GCC_Topography_suite_for_distribution.zip!/GCC_Topography_suite_for_distribution/Helpfiles.docx]

# GCC Topography Suite information

# Summary:

We have developed a suite of programs that enable the fine scale positional mapping of chromosomal interactions. The suite is composed of three programs. The simulation program is of general interest. This program simulates the next generation sequencing of any given genome while incorporating options to fragment (*e.g.* by digestion or random shearing), ligate, shear, purify and sequence the simulated products. The topography program is specific to Chromosome Conformation Capture (3C) and Genome Conformation Capture (GCC) technologies. This program uses either SOAP 1 or BLAST 2 to map sequencing products, which are originally derived from the ligation of two distinct non-adjacent sequences, to a reference genome and output these results for subsequent analyses. The manipulation program enables the user to refine the topology output to levels that are more manageable or significant.

# Introduction:

The next generation sequencing technologies provide powerful tools for analyzing DNA sequences. Although the per-base prices for next-generation sequencing is rapidly dropping it is still an expensive way to perform preliminary experiments to determine necessary read-lengths and coverage levels.

We have developed a suite of programs designed for the Genome Conformation Capture methodology. These programs are required to resolve the sequence data obtained from this proximity based ligation methodology. Moreover, we hope the simulation program will aid biologists as they decide which genome re-sequencing technologies and levels of coverage they require to solve different biological problems. Thus, *in silico* simulations will avoid unnecessary sequence runs and associated financial and time pressures.

The complete tool kit contains 3 programs (*i.e.* Simulation, Topography, and Manipulation). The text below covers the key points for each of these programs and how they are integrated. Help Files are attached in Appendices 1-3.

# The Simulation Program

In the first component of our suite we provide a simulation program to enable the modeling of the sequencing outputs to produce test data. These simulated sequencing runs enable the testing of the various re-sequencing variables (i.e. sequence lengths, paired ends etc…). Moreover, the simulated sequencing data can include GCC data thus enabling the optimization and testing of downstream bioinformatic platforms.

GCC or 3C data is simulated, using the simulation program, by setting restriction site, ligation and sequencing parameters. Alternatively, the simulation program can be used to simply fragment and use next generation sequencing to shot-gun sequence a genome.

# The Topography Program

The second part of our suite (*i.e.* Topography program) is specifically designed to map GCC data, which comprises trans-acting elements in the form of DNA sequences flanking specified restriction sites. GCC is a novel cross-linking dependent ligation approach that relies upon next generation sequencing technologies to map inter- and intra-chromosomal interactions. The topography program cuts sequences at specified restriction sites and uses either the BLAST^8^ or SOAP^7^ algorithm to produce a graph file to enable the user to view these interactions (Figure 1). As such, it provides a way to focus on distinct nodes or regions in the genome.

# The manipulation program.

The manipulation program enables the user to refine the topography output to regions or interaction frequencies, of interest. The output can be viewed on the yED Graph editor (http://yworks.com).

Figure 1: Flow diagram showing the logical information flow through the GCC suite of programs.

# Appendix 1: The simulation program

# 1. Kind of simulation

## 1.1 Fragments against sequences

If this option is ticked you must provide two input sequence files. The first file (a) includes all the sequences and the second file (b) includes sequence fragments. The sequences contained within (b) must be already cut as they will be ligated together with fragments generated by cutting the sequence file (a).

For example:

Sequence file (a):

>seq1

ATGCCCGATATAGAG

>seq2

ATATGGATTATCCCGGGGATATAA

Fragment file (b):

>f1

ATATAGA

>f2

ATATAATTT

If the restriction site is C|CGG (the cleavage is at the position of the vertical bar) the resulting ligated fragments would be (3 ligations per fragment, 30% adjacents, ligation probability = 1, lambda = 1, standard error rate):

>read_0|f1|0|7|seq2|13|24|

atatagacggggatataa

>read_1|f1|0|7|seq2|13|24|

atatagacggggatataa

>read_2|f1|0|7|seq2|13|24|

atatagacgtgggatata

>read_3|f2|0|9|seq2|0|13|seq2|13|24|seq2|0|13|seq2|13|24|

atataGatttatatggattattcccggggatataaatgtggattatcccggggatat

>read_4|f2|0|9|seq2|0|13|

atataatttatatggattatcc

>read_5|f2|0|9|seq2|0|13|

atataatttattatggattatc

Therefore, with these settings the program performs 3 ligations per fragment. Moreover, we obtain a Poisson distributed number of restricted sequences attached to the fragments (read_3 is ligated to 2 segments, see above).

### 1.1.2 Description line of simulation sequence output FASTA header:

>read_5|f2|0|9|seq2|0|13|

1st column is unique sequence identifier: "read"+read number starting at 0

2nd column is the sequence identifier of the query sequence

3rd column is the start of the query sequence

4th column is the stop of the query sequence

5th column same as column 2, but for second restriction fragment

6th column same as column 3, but for second restriction fragment

7th column same as column 4, but for second restriction fragment

In general, except for columns 1-4, the column description is the same as for that in column#-3. This is important where multiple fragments (>2) ligate together.

## 1.2 Only sequences

Here we only have to provide one set of sequences which is later used for processing (digestion, ligation and sequencing).

For example, using the same sequences (sequence file (a), above) and settings (3 ligations per fragment, 30% adjacents, ligation probability = 1, lambda = 1, standard error rate) should produce something like the following:

>read_0|seq1|0|5|seq2|0|13|

atgccatatggattatcc

>read_1|seq1|0|5|seq1|5|16|

atgcccggatatagag

>read_2|seq1|0|5|seq1|5|16|seq1|0|5|

atgcccggaTtatagagatgc

>read_3|seq1|5|16|seq2|13|24|

cggatatagagcggggatataa

>read_4|seq1|5|16|seq2|0|13|seq2|13|24|

cggatatagagatatggattatcccggggatataa

>read_5|seq1|5|16|seq1|0|5|

cggatatagagatgcc

>read_6|seq2|0|13|seq2|13|24|

atatggattatcccggggatataa

>read_7|seq2|0|13|seq1|0|5|seq1|5|16|

atatggattatccatgcccggatatagag

>read_8|seq2|0|13|seq1|0|5|seq2|13|24|

atatggattatccatgcccggggatataa

>read_9|seq2|13|24|seq2|0|13|seq2|13|24|

cggggatataaatatggattatcccggggatataa

>read_10|seq2|13|24|seq2|0|13|seq2|13|24|

cggggatataaatatggattatcccgCgggaataa

>read_11|seq2|13|24|seq1|0|5|seq1|5|16|

cggggatataaatgcccggatatagag

Three reads were created for each segment.

## 2. Upload:

Sequences and sequence fragments can be either pasted into the input box or provided as an input file.

The sequence format can be designated (right hand side) as either:

Raw format:

ATATATATATAGAGAGCCCG

FASTA format:

>seq1 this is a description line

ATAGAGATATAT

>seq2 another unique identifier

ATATATAG

Or GCG format.

## 3. Number of runs:

The number of runs basically specifies the coverage. Therefore, the entire sequencing simulation is repeated as many times as specified under this option.

## 4. Cut DNA on restriction site of choice:

The restriction site should be specified in this field, if the sequences are to be cut (Note: only sequences are cut not fragments). The restriction site has to be in the following format:

C|CGG

The vertical bar indicates the cut position. The program treats the sequence as if it is single stranded and therefore cuts the sequence at the specified position. Restriction sites

are allowed to contain 'N's where N matches any base. However, the sites containing N’s must be symmetrical.

For example, acn|nca or acc|c are allowed but acn|accc is not.

## 5. Ligate (join) fragments together

Fragments can be joined together in various ways. In the preceding examples we only used the first ligation method.

If we join our segments together using this method we progressively go through our segments from 0 until the final segment. Whether each segment ligates to (an)other segment is determined randomly, given the user specified probability. Moreover, the number of ligation products produced is also user specified. Each ligation product consists of one (self-ligations) or more different segments, the number of which is determined from a Poisson distribution with a parameter lambda that is user specified. For non-adjacent ligations the segments which are ligated to each other are chosen randomly out of the segment set. The frequency of adjacent ligations is specified by the user.

To perform a ligation the following parameters must be set:

2) Ligation probability: This must be a value between 0 and 1. If the value is set to one (default) each segment will be ligated to a specified number of other segments (specified by 3). If it is set to zero no ligations will occur and the result file will be empty.
3) Lambda parameter for Poisson distribution: The Poisson distribution=exp(-lambda)*sum_k(lambda^k/k!). If this option is to be used the user should define k.
4) Number of ligations produced per fragment: The user should specify the number of ligation products which are generated for each fragment.
5) Percentage interactions with adjacent fragments: This parameter specifies how often adjacent segments are chosen as interaction partners. For example: segments: s1s2s3s4s5. S1 is to be ligated to 3 different segments. If the proportion of adjacents is set to 33% we would expect on average that one of the ligation products will consist of S1 ligated to the adjacent segment S2. In this case the adjacent set only consists of S2. However, the adjacent set for S2 consists of S1 and S3.

## 6. Provide a defined ligation distribution

The user can provide a distribution file which defines how many segments have how many interactions. This can be used to accurately simulate actual GCC output data, obtained from the topography program’s histogram output.

An example of the histogram file is shown below.

0	0

1	255

2	394

3	656

4	857

5	1018

6	1124

7	1219

8	1148

9	1111

10	980

11	855

12	719

13	583

14	499

15	364

16	305

17	198

18	152

19	104

20	88

21	57

22	43

23	24

24	27

25	13

26	16

In this example, the first column denotes the number of interactions and the second column the number of segments which have that many interactions.

The number of interactions each fragments has is randomly determined. However, the number of interactions each fragment participates in is scored. Thus, if a segment is chosen and this segment was already chosen for and specified as having x interactions, if x has been reached it will be discard. As such, the program will continue choosing 2 random segments till there are no segments left which can interact.

## 7. Provide a number of interactions for a random process ligation

Specify a certain number of interactions where the 2 partners are randomly chosen out of our data set.

## 8. Randomly shear sequences

This step randomly fragments the original sequences, digestion or ligation products (depending on the parameters set at the earlier steps). Input sequences can be randomly sheared using this option to better simulate the sequencing process. The sheared sequences are generated by picking a random position within the sequence and breaking the sequence at fixed intervals to each side. The interval is determined by the mean and the target size range of a normal distribution. Both the mean and target size range are user defined.

## 9. Purify sheared sequences

When the sequences are sheared some fragments will not contain restriction sites. Here the purification efficiency can be set to a value between 0 and 1. The minimal size of the sheared sequences can also be designated.

## 10. Paired end sequencing

The paired end sequencing option enables the sequencing of both ends of the ligation products. The lengths of the sequences are user defined.

## 11. Error rate

The substitution error, insertion error and deletion error can be specified separately under this option. The values are specified as percentages.

## 12. Sequencing of short read sequences

This option enables the generation of short sequences from the end of the digested, ligated or sheared products. Again the length is normally distributed about a used defined mean.

## 13. Kind of output

Here the user specifies whether the output should contain short read sequences or only paired end sequences or both kinds of sequences.

## 15. Output

Here the user specifies the output file name.

## 16. Notes

1) If you want to sequence a restriction map you only need to set the restriction site.
2) For sequencing ligation products there are several ways of producing ligations. a) Provide a number of ligations which occur per fragment and the proportion of adjacent ligations and the probability that those ligations occur. b) Provide a histogram where the number of interactions is relative to the number of fragments. c) Provide the number of desired ligations. In this case the program will produce the number of required ligations by picking the segments, which are ligated, randomly.
3) If the sheared sequences are to be purified, there is also the option to specify the efficiency of purification. The other parameters required are the average sequence length and the range.

# Appendix 2: The Topography program

The topography program imports FASTA formatted simulated or experimentally produced sequence reads. Basically, the topography program reads a FASTA file and sorts sequences containing the nominated restriction site. Sorted sequences are cut at the nominated restriction site and those (and there partners) that are

shorter than a user defined minimum length (default 12 bp) are discarded. The Topography program then uses either BLAST^8^ or SOAP^7^ (which must be installed separately on the host computer) to position the sequence reads onto a reference genome and displays the interactions using a gml output. The program also outputs combined and separate raw graphs, with segment information, for single and multiple hits. It also outputs a Summary file and two histogram outputs.

## 1. File uploads:

### 1.1 Ligation product file:

Either a FASTA file with ligation products or a blast/soap output file. The soap output should be in multiple mode (SOAP parameter –r=2) so that all repeated hits are displayed.

### 1.2 Reference genome sequence:

A reference genome sequence must be provided in FASTA format.

Therefore, the sequence format must look like this:

>ref|unique_name1| description

ATAGAGAG...

>ref|unique_name2| description

AATAGGGA...

"ref" and the vertical bars are required. The unique_names must correspond to the LOCUS name in the GenBank files. The user should specify if the data that is provided is simulated or real. If the data is simulated (using the Simulation program) the topography program will identify which proportion of the reads are correctly matched back to the reference genome.

### 1.3 GenBank file with genome positioning information:

This information is required to provide annotations for the nodes in the graph files. Genome positioning information must be provided in a GenBank format (www.ebi.ac.uk/embl/Documentation/FT_definitions/feature_table.html) and the annotation file should look like this:

```
LOCUS       NC_001133             230208 bp    DNA     linear   PLN 11-FEB-2008
DEFINITION  Saccharomyces cerevisiae chromosome I, complete chromosome
            sequence.
ACCESSION   NC_001133
VERSION     NC_001133.7  GI:144228165
KEYWORDS    .
SOURCE      Saccharomyces cerevisiae (baker's yeast)
  ORGANISM  Saccharomyces cerevisiae
            Eukaryota; Fungi; Ascomycota; Saccharomycotina; Saccharomycetes;
            Saccharomycetales; Saccharomycetaceae; Saccharomyces.
REFERENCE   1  (bases 1 to 230208)
  AUTHORS   Goffeau,A., Barrell,B.G., Bussey,H., Davis,R.W., Dujon,B.,
            Feldmann,H., Galibert,F., Hoheisel,J.D., Jacq,C., Johnston,M.,
            Louis,E.J., Mewes,H.W., Murakami,Y., Philippsen,P., Tettelin,H. and
            Oliver,S.G.
  TITLE     Life with 6000 genes
  JOURNAL   Science 274 (5287), 546-547 (1996)
   PUBMED   8849441
REFERENCE   2  (bases 1 to 230208)
  AUTHORS   Bussey,H., Kaback,D.B., Zhong,W., Vo,D.T., Clark,M.W., Fortin,N.,
            Hall,J., Ouellette,B.F., Keng,T., Barton,A.B. et al.
  TITLE     The nucleotide sequence of chromosome I from Saccharomyces
            cerevisiae
  JOURNAL   Proc. Natl. Acad. Sci. U.S.A. 92 (9), 3809-3813 (1995)
   PUBMED   7731988

REFERENCE   3  (bases 1 to 230208)
  CONSRTM   NCBI Genome Project
  TITLE     Direct Submission
  JOURNAL   Submitted (11-FEB-2008) National Center for Biotechnology
            Information, NIH, Bethesda, MD 20894, USA
REFERENCE   4  (bases 1 to 230208)
  AUTHORS   Saccharomyces Genome Database (yeast-curator@genome.stanford.edu).
  TITLE     Direct Submission
  JOURNAL   Submitted (17-NOV-1999) Department of Genetics, Stanford
            University, Saccharomyces Genome Database, Stanford, CA 94305-5120,
            USA
COMMENT     REVIEWED REFSEQ: This record has been curated by SGD.
FEATURES             Location/Qualifiers
     source          1..230208
                     /organism="Saccharomyces cerevisiae"
                     /mol_type="genomic DNA"
                     /strain="S288C"
                     /db_xref="taxon:4932"
                     /chromosome="I"
     repeat_region   complement(1..801)
                     /gene="TEL01L"
                     /note="Telomeric region on the left arm of Chromosome I;
                     composed of an X element core sequence, X element
                     combinatorial repeats, and a short terminal stretch of
                     telomeric repeats"
                     /inference="non-experimental evidence, no additional
                     details recorded"
                     /rpt_family="Telomeric Region"
                     /db_xref="SGD:S000028862"
     repeat_region   complement(1..62)
                     /gene="TEL01L-TR"
                     /note="Terminal stretch of telomeric repeats on the left
                     arm of Chromosome I"
                     /inference="non-experimental evidence, no additional
                     details recorded"
                     /rpt_family="Telomeric Repeat"
                     /db_xref="SGD:S000028864"
     repeat_region   complement(63..336)
                     /gene="TEL01L-XR"
                     /note="Telomeric X element combinatorial Repeat region on
                     the left arm of Chromosome I; contains repeats of the D,
                     C, B and A types, as well as Tbf1p binding sites; formerly
                     called SubTelomeric Repeats"
                     /inference="non-experimental evidence, no additional
ORIGIN
        1 ccacaccaca cccacacacc cacacaccac accacacacc acaccacacc cacacacaca
       61 catcctaaca ctaccctaac acagccctaa tctaaccctg gccaacctgt ctctcaactt
      121 accctccatt accctgcctc cactcgttac cctgtcccat tcaaccatac cactccgaac
      181 caccatccat ccctctactt actaccactc acccaccgtt accctccaat tacccatatc
      241 caacccactg ccacttaccc taccattacc ctaccatcca ccatgaccta ctcaccatac
      301 tgttcttcta cccaccatat tgaaacgcta acaaatgatc gtaaataaca cacacgtgct
      361 taccctacca ctttatacca ccaccacatg ccatactcac cctcacttgt atactgattt
      421 tacgtacgca cacggatgct acagtatata ccatctcaaa cttaccctac tctcagattc
      481 cacttcactc catggcccat ctctcactga atcagtacca aatgcactca catcattatg
      541 cacggcactt gcctcagcgg tctataccct gtgccattta cccataacgc ccatcattat
      601 ccacattttg atatctatat ctcattcggc ggtcccaaat attgtataac tgcccttaat
      661 acatacgtta taccactttt gcaccatata cttaccactc catttatata cacttatgtc
      721 aatattacag aaaaatcccc acaaaaatca cctaaacata aaaatattct acttttcaac
      781 aataatacat aaacatattg gcttgtggta gcaacactat catggtatca ctaacgtaaa
      841 agttcctcaa tattgcaatt tgcttgaacg gatgctattt cagaatattt cgtacttaca
      901 caggccatac attagaataa tatgtcacat cactgtcgta acactcttta ttcaccgagc
      961 aataatacgg tagtggctca aactcatgcg ggtgctatga tacaattata tcttatttcc
```

## 2. Search result or FASTA file

Specify whether the ligation product file was in FASTA or search result format.

## 3. Sequence properties

### 3.1 Restriction site

To cut the ligation products within the FASTA file the restriction site must be specified here: The restriction site must be in the following format:

C|CGG

Where the vertical bar indicates the cut position. For the restriction component, the program treats the sequence as if it is single stranded and therefore cuts the sequence at the specified position. Restriction sites are allowed to contain 'N's where N matches any base. However, the sites containing N’s must be symmetrical.

For example, acn|nca or acc|c are allowed but acn|accc is not.

### 3.2 Minimum sequence length:

This parameter determines the minimum sized sequence that will be used by the positioning algorithms to query the genome sequence. It refers to the minimum sequence length on either side of the restriction site. All sequences that contain a restriction fragment, defined by the nominated restriction site (3.1), which is smaller than the specified size are discarded. Maximum sequence length,

sequences which are longer than the specified maximum size are cut off and only the sequence up to the maximum length are used for the sequence search.

## 4. Program

This option designates the program which was or is to be used to produce the search result file. The two options are BLAST^8^ or SOAP^7^.

## 5. Output files

### 5.1 GML output:

GML outputs can be used as input file for graph viewing programs like yED.

### 5.2 Graph output file:

A raw graph output which can be used as input for the manipulation program or for further analyses.

This is a tab delimited file of the following format:

2	3	3

3	186	1

3	2	3

3	4	2

3	13241	2

3	1160	3

3	12500	1

3	10351	1

4	3	2

4	3492	1

4	5	8

5	13608	1

5	4	8

5	5	2

5	6	4

6	5	4

9	1356	1

9	10	1

9	13561	1

10	9	1

10	11	1

11	888	1

11	10	1

31	32	2

The first column denotes node1, the second column node2 and the third column the number of sequences that represented interactions between node1 and node2. Three files are output at this point: a) the unique file which includes all the nodes which are positioned at a single locus in the reference genome. This file is designated .dat. b) The file which ends with "mult" contains information on all the interactions that result from sequences which hit the reference genome multiple times; and c) The .comb file. The .comb file contains information on all the interactions present in the data-set irrespective of whether they are unique or repetitive.

### 5.3 Histogram output:

This file provides a distribution of how many interactions the nodes(segments) are involved in.

The file format looks like this:

0	0

1	255

2	394

3	656

4	857

5	1018

6	1124

7	1219

8	1148

9	1111

10	980

11	855

12	719

13	583

14	499

15	364

16	305

17	198

18	152

19	104

20	88

21	57

22	43

23	24

24	27

25	13

26	16

27	7

28	8

29	9

30	4

31	3

32	4

33	2

34	3

35	4

36	2

The first column denotes the number of interactions and the second column denotes the number of nodes which have that many interactions. In the example above, 2 nodes are interacting 36 times. Again there are 3 files output. The file without any additional extensions is the single hit output, the output with the "mult" extension is the output for everything which is involved with multiple hits, and the "comb" extension file is the first two outputs combined.

### 5.4 Skyline output:

For the skyline output all segments (or genomic sequences) are concatenated together. The values in the first column denote the position of the segment of interest within the genome and the second column denotes the number of interaction for this segment.

The output is in the following tab-delimited format:

1889	0

1925	0

3988	3

6436	13

7763	11

11735	15

14836	4

15348	0

15543	0

17809	3

19475	2

19745	2

20684	0

21663	0

22590	0

24050	0

24167	0

25496	0

25589	0

25724	0

25859	0

25994	0

26129	0

26264	0

26399	0

26534	0

26669	0

26804	0

27074	0

27116	0

28621	2

29173	4

29479	12

30894	9

31685	7

32138	8

32148	0

Again there are three output files differing in the presence or absence of no or the "mult" or "comb extension. There is also a normalized output which normalizes the number of interactions to interactions per 100 base pairs. But this output is only available for the single hits and is denoted by the “100” extension.

### 5.5 Summary output:

The summary ouput file lists the hits of the reads against the reference genome and when a genomic map is provided it puts the segment number in the 3rd column. At the end of the file a summary about the single, multiple and correct hits is provided.

# Appendix 3: The Manipulation program:

The manipulation program reads a raw graph in the following format node1\tnode2\t#interactions and outputs a gml graph based on the numbers of #interactions, #repeated interactions, or position specific information.

## 3.1 Genome positioning information (GenBank file):

This information is required to provide annotations for the nodes in the graph files. Genome positioning information must be provided in a GenBank format (www.ebi.ac.uk/embl/Documentation/FT_definitions/feature_table.html) and the annotation file should look like this:

```
LOCUS       NC_001133             230208 bp    DNA     linear   PLN 11-FEB-2008
DEFINITION  Saccharomyces cerevisiae chromosome I, complete chromosome
            sequence.
ACCESSION   NC_001133
VERSION     NC_001133.7  GI:144228165
KEYWORDS    .
SOURCE      Saccharomyces cerevisiae (baker's yeast)
  ORGANISM  Saccharomyces cerevisiae
            Eukaryota; Fungi; Ascomycota; Saccharomycotina; Saccharomycetes;
            Saccharomycetales; Saccharomycetaceae; Saccharomyces.
REFERENCE   1  (bases 1 to 230208)
  AUTHORS   Goffeau,A., Barrell,B.G., Bussey,H., Davis,R.W., Dujon,B.,
            Feldmann,H., Galibert,F., Hoheisel,J.D., Jacq,C., Johnston,M.,
            Louis,E.J., Mewes,H.W., Murakami,Y., Philippsen,P., Tettelin,H. and
            Oliver,S.G.
  TITLE     Life with 6000 genes
  JOURNAL   Science 274 (5287), 546-547 (1996)
   PUBMED   8849441
REFERENCE   2  (bases 1 to 230208)
  AUTHORS   Bussey,H., Kaback,D.B., Zhong,W., Vo,D.T., Clark,M.W., Fortin,N.,
            Hall,J., Ouellette,B.F., Keng,T., Barton,A.B. et al.
  TITLE     The nucleotide sequence of chromosome I from Saccharomyces
            cerevisiae
  JOURNAL   Proc. Natl. Acad. Sci. U.S.A. 92 (9), 3809-3813 (1995)
   PUBMED   7731988
REFERENCE   3  (bases 1 to 230208)
  CONSRTM   NCBI Genome Project
  TITLE     Direct Submission
  JOURNAL   Submitted (11-FEB-2008) National Center for Biotechnology
            Information, NIH, Bethesda, MD 20894, USA
REFERENCE   4  (bases 1 to 230208)
  AUTHORS   Saccharomyces Genome Database (yeast-curator@genome.stanford.edu).
  TITLE     Direct Submission
  JOURNAL   Submitted (17-NOV-1999) Department of Genetics, Stanford
            University, Saccharomyces Genome Database, Stanford, CA 94305-5120,
            USA
COMMENT     REVIEWED REFSEQ: This record has been curated by SGD.
FEATURES             Location/Qualifiers
     source          1..230208
                     /organism="Saccharomyces cerevisiae"
                     /mol_type="genomic DNA"
                     /strain="S288C"
                     /db_xref="taxon:4932"
                     /chromosome="I"
     repeat_region   complement(1..801)
                     /gene="TEL01L"
                     /note="Telomeric region on the left arm of Chromosome I;
                     composed of an X element core sequence, X element
                     combinatorial repeats, and a short terminal stretch of

                     telomeric repeats"
                     /inference="non-experimental evidence, no additional
                     details recorded"
                     /rpt_family="Telomeric Region"
                     /db_xref="SGD:S000028862"
     repeat_region   complement(1..62)
                     /gene="TEL01L-TR"
                     /note="Terminal stretch of telomeric repeats on the left
                     arm of Chromosome I"
                     /inference="non-experimental evidence, no additional
                     details recorded"
                     /rpt_family="Telomeric Repeat"
                     /db_xref="SGD:S000028864"
     repeat_region   complement(63..336)
                     /gene="TEL01L-XR"
                     /note="Telomeric X element combinatorial Repeat region on
                     the left arm of Chromosome I; contains repeats of the D,
                     C, B and A types, as well as Tbf1p binding sites; formerly
                     called SubTelomeric Repeats"
                     /inference="non-experimental evidence, no additional
ORIGIN
        1 ccacaccaca cccacacacc cacacaccac accacacacc acaccacacc cacacacaca
       61 catcctaaca ctaccctaac acagccctaa tctaaccctg gccaacctgt ctctcaactt
      121 accctccatt accctgcctc cactcgttac cctgtcccat tcaaccatac cactccgaac
      181 caccatccat ccctctactt actaccactc acccaccgtt accctccaat tacccatatc
      241 caacccactg ccacttaccc taccattacc ctaccatcca ccatgaccta ctcaccatac
      301 tgttcttcta cccaccatat tgaaacgcta acaaatgatc gtaaataaca cacacgtgct
      361 taccctacca ctttatacca ccaccacatg ccatactcac cctcacttgt atactgattt
      421 tacgtacgca cacggatgct acagtatata ccatctcaaa cttaccctac tctcagattc
      481 cacttcactc catggcccat ctctcactga atcagtacca aatgcactca catcattatg
      541 cacggcactt gcctcagcgg tctataccct gtgccattta cccataacgc ccatcattat
      601 ccacattttg atatctatat ctcattcggc ggtcccaaat attgtataac tgcccttaat
      661 acatacgtta taccactttt gcaccatata cttaccactc catttatata cacttatgtc
      721 aatattacag aaaaatcccc acaaaaatca cctaaacata aaaatattct acttttcaac
      781 aataatacat aaacatattg gcttgtggta gcaacactat catggtatca ctaacgtaaa
      841 agttcctcaa tattgcaatt tgcttgaacg gatgctattt cagaatattt cgtacttaca
      901 caggccatac attagaataa tatgtcacat cactgtcgta acactcttta ttcaccgagc
      961 aataatacgg tagtggctca aactcatgcg ggtgctatga tacaattata tcttatttcc
```

## 3.2 Reference genome sequence:

A reference genome sequence must be provided in FASTA format.

Therefore, the sequence format should look like this:

>ref|unique_name1| description

ATAGAGAG...

>ref|unique_name2| description

AATAGGGA...

"ref" and the vertical bars are required. The unique_names must correspond to the LOCUS name in the GenBank files. The user should specify if the data that is

provided is simulated or real. If the data is simulated (using the Simulation program) the topography program will identify which proportion of the reads are correctly matched back to the reference genome.

## 3.3 Input graph file:

The expected input is a graph output file from the topography program. The file should be in the following format:

2	3	3

3	186	1

3	2	3

3	4	2

3	13241	2

3	1160	3

3	12500	1

3	10351	1

4	3	2

4	3492	1

4	5	8

5	13608	1

5	4	8

5	5	2

5	6	4

6	5	4

9	1356	1

9	10	1

9	13561	1

10	9	1

10	11	1

11	888	1

11	10	1

31	32	2

The first column denotes node1, the second column node2 and the third column the number of observed interactions between node1 and node2. Other columns

which may contain additional information about the nodes or the interaction are simply ignored by the program.

## 3.4 Restriction site:

The restriction site must be in the following format:

C|CGG

The vertical bar indicates the cut position. The program treats the sequence as if it is single stranded and therefore cuts the sequence at the specified position. Restriction sites are allowed to contain 'N's where N matches any base. However, the sites containing N’s must be symmetrical.

For example, acn|nca or acc|c are allowed but acn|accc is not.

## 3.5. Position file:

If a graph is required which only contains information on certain regions of the genome it is possible to provide a position file.

The position file should be tabbed delimited and in the following format:

NC_001138 12663 100000

NC_001137 3333 5454

The first column defines the unique sequence identifier from the genome reference FASTA file. The second column defines the start position and the third column the end position of the sequence of interest. If this option is used only nodes which fall into those regions or nodes which interact with nodes which fall into those regions are output.

## 3.6 Graph restrictions:

### 3.6.1 Minimum number of interactions per node.

If the "Minimum number of interactions per node" field is set to n. Only nodes which are involved in greater or equal than n interactions are displayed.

### 3.6.2 Minimum number of interactions between the same partners.

If the "Minimum number of interactions between the same partners" field is set to n greater than 0, only nodes which have at least 1 partner with which they are interacting n or greater times are output.

### 3.6.3 Maximum number of interactions per node.

If the "Maximum number of interactions per node" field is set to n other than max only nodes which are involved in less or equal than n interactions are displayed.

### 3.6.4 Maximum number of interactions between the same partners.

If the "Maximum number of interactions with the same partner" field is set to n other than max, only interactions and nodes associated with those interactions which are repeated less than n times are displayed.

## 7. Output files:

### 7.1 Output graph file in gml format:

This file can serve as an input file for graph viewing programs like yED and serves to provide an overview of the data.

### 7.2 Output FASTA file for segments without interactions:

This option outputs all of the sequences which are involved in no interactions. This output includes the short reads which could not be used for a sequence search.

### 7.3 Output FASTA file for segments which fulfill the specified restrictions:

This option produces a FASTA file of the segments which fulfill the specified restrictions. This includes the sequences of all the segments which interact as nodes in gml graph file.

### 7.4 Reanalysis: no N’s

Please note that Topography allows one mismatch as default. In order to run with no mismatches you need to follow the instructions below. Sorry we never went back to change the hard code. I have included an example for E. coli that we have recently run.

The topography program does not take parameters for SOAP so the .outtemp files that are produced need to be copied and run through SOAP with the extra parameter of -f 0 to exclude any sequences that contine Ns.

To achieve this the .outtemp files from the first Topography run for are copied to a new folder.

Remove the .fas.out files.

Set up the following bash scripts to re-run SOAP with the altered parameters. SOAP instructions were obtained from the “.javaerr” and modifed with the extra parameters: -f 0 to exclude sequences with Ns, -p 3 to run SOAP on three processors, -s was changed to match that given in the “.javaerr” output file, and the directories were changed to work on the “copy.outtemp” folder:

#!/bin/bash

soap -w 5 -f 0 -p 3 -v 0 -r 1 -s 5 -o /copy.outtemp/lensorted13.fas.out -d /Genome_sequences/Genome_sequences_for_mapping_with_ligationcontrols/genome_with_controls.fas -a /copy.outtemp/lensorted13.fas

Once the bash scripts were made they were made exacutable by:

$ chmod +x filename.sh

The scripts were run as:

$ bash filename.bch

Once SOAP had been run the files needed to be sorted, here is an example script for E. coli sample L (combined_L1_L2):

sort -n -m /scratch/E_coli_MG1655_GCC/Interlaced_files/combined_L1_L2/combined_L1_L2_copy.outtemp/lensorted13.fas.out /scratch/E_coli_MG1655_GCC/Interlaced_files/combined_L1_L2/combined_L1_L2_copy.outtemp/lensorted14.fas.out /scratch/E_coli_MG1655_GCC/Interlaced_files/combined_L1_L2/combined_L1_L2_copy.outtemp/lensorted15.fas.out /..................

/scratch/E_coli_MG1655_GCC/Interlaced_files/combined_L1_L2/combined_L1_L2_copy.outtemp/lensorted98.fas.out /scratch/E_coli_MG1655_GCC/Interlaced_files/combined_L1_L2/combined_L1_L2_copy.outtemp/lensorted99.fas.out /scratch/E_coli_MG1655_GCC/Interlaced_files/combined_L1_L2/combined_L1_L2_copy.outtemp/lensorted100.fas.out -o /scratch/E_coli_MG1655_GCC/Interlaced_files/combined_L1_L2/L_ra_13-100.searchout -T /scratch/E_coli_MG1655_GCC/Interlaced_files/combined_L1_L2

Once “sort” had been run “topography_1.19.jar” was re-run using the “.searchout” file generated by the “sort” run. First a topography shell script was made and saved as “filename.sh”.

#!/bin/bash

TOPO="/jar/topography_1.19.jar"

java -jar -Xmx8000m -jar $TOPO \

--use_gui "false" \

-i "/scratch/E_coli_MG1655_GCC/Interlaced_files/combined_L1_L2/L_ra_13-100.searchout" \

-i2pe "" \

-d "/ /Genome_sequences/Genome_sequences_for_mapping_with_ligationcontrols/genome_with_controls.fas" \

-gbk "/Genome_sequences/Genome_sequences_for_mapping_with_ligationcontrols/GBK_with_controls.gbk" \

--soapsplit_d "" \

--real_or_sim "real" \

--do_blast "false" \

--restriction "gcg|c" \

--minlen "13" \

--maxlen "90" \

--paired "true" \

--parser_tp "slim" \

--program "soap" \

--processors "2" \

--minlen_1mm "9999" \

-o "combined_L1_L2/L_ra_13-100" \

> combined_L1_L2/L_ra_13-100.out 2> combined_L1_L2/L_ra_13-100.err

The file was then made exacutable by “$ chmod +x filename.sh”:

$ chmod +x filename.sh

Topography was then run:

$ sh filename.sh

**References**

^1^	Li, R., Li, Y., Kristiansen, K., & Wang, J., SOAP: short oligonucleotide alignment program. *Bioinformatics* 24 (5), 713-714 (2008).

^2^	Altschul, S.F., Gish, W., Miller, W., Myers, E.W., & Lipman, D.J., Basic local alignment search tool. *J Mol Biol* 215 (3), 403-410 (1990).
