# Supplementary material for: Chromosome conformation maps in fission yeast reveal cell cycle dependent sub nuclear structure
Source: Nucleic Acids Res. 2014 Oct 23;42(20):12585–99. doi: 10.1093/nar/gku965 (PMC4227791; doi:10.1093/nar/gku965)
Supplement: SUPPLEMENTARY DATA [file supp_gku965_nar-01186-n-2014-File009.zip › GCC_Topography_suite_for_distribution.zip!/GCC_Topography_suite_for_distribution/Simulator FAQs.docx]

```
1) The software won’t run.

Answer: You need the latest version of java 1.6.013 or better (Java(TM) SE Runtime Environment (build 1.6.0_13-b03) Java HotSpot(TM) Client VM (build 11.3-b02, mixed mode, sharing)).

2) I get the following error when I try to run the software in OS X terminal and linux:

 >$ java -version
java version "1.5.0_19"
Java(TM) 2 Runtime Environment, Standard Edition (build 1.5.0_19-
b02-304)
Java HotSpot(TM) Client VM (build 1.5.0_19-137, mixed mode, sharing)  >$ java -jar simulation.jar Exception in thread "main" java.lang.UnsupportedClassVersionError: Bad version number in .class file
	at java.lang.ClassLoader.defineClass1(Native Method)
	at java.lang.ClassLoader.defineClass(ClassLoader.java:675)
	at java.security.SecureClassLoader.defineClass(SecureClassLoader.java:
124)
	at java.net.URLClassLoader.defineClass(URLClassLoader.java:260)
	at java.net.URLClassLoader.access$100(URLClassLoader.java:56)
	at java.net.URLClassLoader$1.run(URLClassLoader.java:195)
	at java.security.AccessController.doPrivileged(Native Method)
	at java.net.URLClassLoader.findClass(URLClassLoader.java:188)
	at java.lang.ClassLoader.loadClass(ClassLoader.java:316)
	at sun.misc.Launcher$AppClassLoader.loadClass(Launcher.java:280)
	at java.lang.ClassLoader.loadClass(ClassLoader.java:251)
	at java.lang.ClassLoader.loadClassInternal(ClassLoader.java:374)

Answer: This is a problem with the Java version. You need to have java 1.6.013 or later.

3) Does this program only run through a GUI?

Answer: Yes. The program runs as a GUI. However, when you start it in Linux you can allocate it more memory i.e.  java - Xmx1500m -jar

4) Does it run in Windows?

Answer: Yes, run it from command line. Allocate more memory to the java program see 2) above.

5) 	What does the number of runs mean?

Kind of simulation:			Only sequences
Number of runs:				30
Randomly shear sequences:	On
Target size mean:			300
Target size range:			0
Paired end sequencing:		Whole sequence

I take it that the 'number of runs' box is actually for some value
that is proportional to genome coverage -- here, 30-fold coverage.

Answer: Yes this is the number of runs the software does, for example 30 means it will run the simulation on the sample 30 times.

6) Incidentally, is there a 2-fold error here?  For a genome size of 5500 and a read size of 75-bp, I got 1080 reads in total (i.e., reads, not pairs of reads).  This is only ~15-fold coverage.

Answer: The target size is the total fragment size, hence 300 bp fragments are produced from this run. But the Paired end sequences were set for 75-bp fragments therefore you will only sequence 150-bp of each fragment, i.e. 50%.
```
